# Preclinical evidence of the effect of icariin on diabetic nephropathy: a systematic review and meta-analysis

**DOI:** 10.1186/s13098-025-01760-2

**Published:** 2025-06-18

**Authors:** Xueli Man, Peiyao Ren, Juan Jin, Qiang He

**Affiliations:** Department of Nephrology, The First Affiliated Hospital of Zhejiang Chinese Medical University (Zhejiang Provincial Hospital of Chinese Medicine), Hangzhou, 310000 Zhejiang China

**Keywords:** Icariin, Diabetic nephropathy, Animal experiment, Meta-analysis, Systematic review

## Abstract

**Background:**

Icariin (ICA), a bioactive flavonoid derived from Epimedium species, has demonstrated anti-inflammatory and anti-fibrotic properties in preclinical studies, suggesting potential therapeutic effects on diabetic nephropathy (DN). However, systematic evaluation of its efficacy remains unclear.

**Objective:**

The purpose of this study is to evaluate the efficacy of Icariin on DN by preclinical evidence and meta-analysis. Meanwhile, the main possible action mechanisms of Icariin against DN were also summarized.

**Methods:**

As of October 1, 2024, we conducted a systematic search across seven prominent Chinese and English databases (CNKI, Wanfang, CBM, PubMed, Cochrane Library, Embase, and Web of Science) to identify studies investigating the therapeutic effects of icariin on DN. PROSPERO has released a summary protocol (registration number: CRD42024564001).

**Results:**

This meta-analysis encompassed nine studies, involving a total of 308 animals, and revealed that icariin significantly reduced blood glucose, SCR, BUN, 24 h UP, 24 h UV, KI, MDA, and IL-1β levels, while augmenting antioxidant enzyme activities (SOD and GPX). Furthermore, ICA lowered TG and TC, indicative of its potential in mitigating risk factors. However, direct comparisons between ICA and angiotensin II receptor blockers (ARB) yielded no statistically significant differences in DN treatment outcomes (p > 0.05). The greatest effects were recorded in high-dose (> 30 mg/kg/day) groups rather than in low-dose (< 30 mg/kg/day) groups. For time-response effects, subgroup analysis indicated that intervention duration of ICA can influence the treatment effect, and more beneficial effects were observed when studies had a drug administration time of < 8 weeks.

**Conclusion:**

Based on an analysis of existing experimental evidence, icariin displays promise in slowing the progression of diabetic nephropathy. To validate its anti-diabetic nephropathy efficacy with greater precision and ensure its readiness for clinical translation, further confirmatory animal studies are warranted.

**Supplementary Information:**

The online version contains supplementary material available at 10.1186/s13098-025-01760-2.

## Highlights

Icariin demonstrates a potential in mitigating the onset and progression of diabetic nephropathy, attributed to its multifaceted pharmacological actions such as antioxidant, antifibrotic, antiapoptotic, and anti-inflammatory properties; Conducting rigorous preclinical systematic evaluations is instrumental in enhancing the overall quality and reliability of animal experimentation pertaining to these therapeutic effects.

**Supplementary Information:**

The online version contains supplementary material available at 10.1186/s13098-025-01760-2.

## Background

DN stands as a critical complication of diabetes, contributing significantly to the burden of end-stage renal disease. Prior research has implicated multiple factors in DN pathogenesis, such as advanced glycation product accumulation [1], hemodynamic alterations [2], hormonal imbalances [3], and their sequelae—a cascade of excessive inflammation [4], oxidative stress [5] and interstitial fibrosis [6]. Clinical manifestations encompass persistent proteinuria, declining glomerular filtration rate, and abnormal blood pressure [7], while typical pathological findings reveal mesangial matrix expansion, basement membrane thickening, and renal tubular atrophy [8]. Amidst this backdrop, the global prevalence of diabetes and its associated complications continues to escalate [9]. Projections indicate that by 2045, approximately 783.2 million individuals may be afflicted with diabetes [10], with a substantial proportion—ranging from 25 to 40% in T1DM and 5% to 40% in T2DM patients [11]—ultimately progressing to DN. Contemporary DN management relies heavily on antihypertensive agents (e.g., ACEi, ARB) [12], innovative hypoglycemic therapies (GLP-1 receptor agonists, SGLT2 inhibitors) [13], and mineralocorticoid receptor antagonists (MRA) [14]. Nevertheless, these treatments have limitations in halting the progression towards ESRD, constraining their clinical utility and posing significant challenges to patient quality of life [15]. Given the treatment dilemma surrounding DN, there is an urgent need to develop novel, more effective, and safer therapeutic options.

**Fig. 1:**
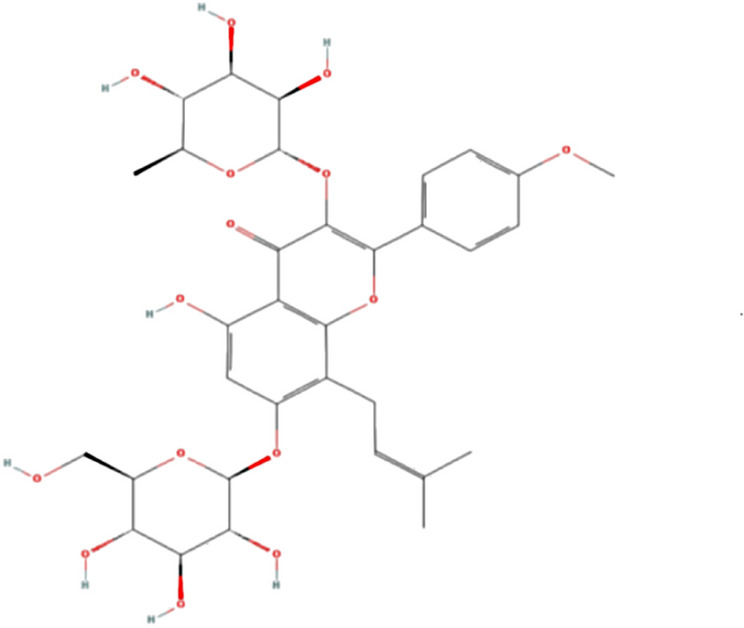
The chemical structure of icariin

In recent years, traditional Chinese medicine (TCM) has emerged as a viable therapeutic approach for DN. Among these, Herba Epimedii, commonly known as Xianling pi, holds a rich historical significance in Chinese medicine, renowned for its ability to nourish the kidney, strengthen muscles and bones, and replenish yin energy [16]. With its high medicinal potential, Herba Epimedii has garnered interest from both Eastern and Western medical communities. Prior research underscores its efficacy in addressing diverse health conditions, including osteoporosis, ischemia, cancer, liver protection, neuroprotection, antidepressant effects, and antioxidant stress reduction [17–19]. Icariin(C41H68O14, MW: 676.67, Fig. 1), the primary bioactive component of Epimedium [20], has been extensively studied for its multifaceted pharmacological actions in DN management, encompassing anti-fibrotic, antioxidant, anti-inflammatory, anti-apoptotic properties, as well as modulation of glucose/lipid metabolism and immune function [21–23]. However, there are few reports on the clinical use of icariin in the treatment of diabetic nephropathy, and the related clinical studies can not be analyzed by Meta. Fortunately, there has been a number of preclinical evidence that icariin has an effect on DN. Therefore, we collected data from animal studies for systematic review and meta-analysis, and analyzed the protective effect and mechanism of icariin in DN animal model by minimizing the deviation of results. We believe that the construction of evidence-based experiments at the animal level will help to apply preclinical findings to the clinical environment and provide support for the development and transformation of icariin.

## Methods

We designed this meta analysis according to the PRISMA table of the preferred reporting item to evaluate the effect of icariin on diabetic nephropathy. PROSPERO has released a summary protocol (Registration number: CRD42024564001).

### Literature search strategies

To comprehensively assess the literature on icariin's therapeutic potential in DN, we conducted a systematic search across key Chinese and English databases, including PubMed, Web of Science, Embase, Cochrane, Wanfang, CNKI, and the China Biomedical Database, spanning from the inception of these databases up to September 1st, 2024. The search strategy encompassed both medical subject headings (MeSH) and relevant free-text terms to ensure a comprehensive capture of all relevant articles. The search terms were expressed as follows: ‘‘Icariin’’, ‘‘Herba epimedii’’, ‘‘Xianling pi’’, ‘‘diabetic nephropathy’’, ‘‘diabetic kidney disease’’, ‘‘diabetic glomerulosclerosis’’, ‘‘end-stage renal disease’’.

1. Diabetic Nephropathies.
2. Diabetic Kidney Disease.
3. Diabetic glomerulopathy.
4. End stage renal disease.
5. OR 2 OR 3 OR 4
6. Icariin.
7. Herba epimedii.
8. Xianling Pi.
9. OR 7 OR 8
10. AND 9

### Eligibility criteria

Inclusion criteria for the study were: (1) Experimental groups comprising diabetic animals treated with icariin, (2) Control groups of diabetic animals without any drug intervention, (3) Randomized controlled trials involving animals without restrictions on age, sex, breed, or induction methods, (4) Studies with clear endpoint definitions and complete data sets, and (5) No language limitations were imposed on the included studies.

Exclusion criteria encompassed: (1) Non-animal experiments, such as cell-based studies or human trials, (2) Studies lacking a randomized controlled design, including case studies, crossover, and cross-sectional designs, (3) Non-original research articles, for instance, reviews, editorials, conference abstracts, case reports, and meta-analyses, (4) Studies without clear outcome indicators, (5) Duplicate or inaccessible data, (6) Studies that failed to report sample size (N) or provide sufficient data type information, such as standard deviation (SD) or standard error (SE), (7) Ambiguous results (e.g., unreported standard deviations, undefined clinical endpoints), and (8) Incomplete data (e.g., missing baseline characteristics, dropout rates).

### Data extraction and quality assessment

Articles that did not meet the inclusion and exclusion criteria were excluded. Subsequently, two independent reviewers assessed the quality of the remaining articles and extracted pertinent data, encompassing general information (first author and publication year), animal characteristics (age, species, sex, body weight, sample size, diabetes induction method), icariin administration details (route, dose, timing, frequency), and primary outcomes (blood glucose, SCR, BUN, 24 h UP, 24 h UV, KI) alongside secondary outcomes (MDA, GPX, SOD, IL-1β, TG, TC). All data for meta-analysis were verified to include animal sample size (N), mean values, and either SD or SE of the mean. In cases of discrepancies during data extraction, consensus was achieved through consultation with a third reviewer. For data presented in graphical form, efforts were made to contact the authors for the original data. If unsuccessful, WebPlotDigitizer 4.5 software was utilized to extract the data. All reported SE were converted to SD using the formula SD = SE × √N [24]. For studies reporting outcomes at multiple time points, only data from the final time point were included in the meta-analysis. Furthermore, data from all subgroups were extracted to ensure a comprehensive analysis.

Two independent researchers (MXL and RPY) conducted a bias risk assessment of the included studies utilizing the SYRCLE Animal Study Bias Risk Tool [25]. This assessment encompassed ten criteria: (1) sequence generation, (2) baseline comparability, (3) allocation concealment, (4) random housing, (5) blinding of caregivers and investigators, (6) random outcome assessment, (7) blinding of outcome assessors, (8) incomplete outcome data, (9) selective outcome reporting, and (10) other potential biases. Each criterion was categorized as low, unclear, or high risk. Any discrepancies arising during the quality appraisal were resolved through discussion with a third reviewer (JJ).

### Statistical analysis

For data extraction and analysis, WebPlotDigitizer (version 4.7) and RevMan (version 5.4) were employed, while descriptive statistics were utilized to characterize the study population. When comparing results across varying measurement methods or scales, the standardized mean difference (SMD) was adopted, with a 95% confidence interval. Statistical significance was set at P < 0.05. To assess statistical heterogeneity, I^2^ was calculated, and values exceeding 50% were considered indicative of significant heterogeneity. Given the exploratory nature of animal studies, random effects models were applied to account for anticipated heterogeneity. Funnel plots were used to assess potential publication bias for outcome measures. To investigate potential sources of heterogeneity across studies, we performed subgroup analyses focusing on the four primary outcomes with sufficient research data: RBG, SCR, BUN, and KI. These analyses were stratified by treatment duration (≤ 8 weeks and > 8 weeks) and icariin dosage (≤ 30 mg/kg, 30–80 mg/kg, and ≥ 80 mg/kg). Statistical significance was deemed present at a P-value threshold of < 0.05. Sensitivity analysis employing meta-based influence assessment is conducted to mitigate the potential impact of small sample sizes and to ascertain the robustness of the findings.

## Results

### Study selection

**Fig. 2:**
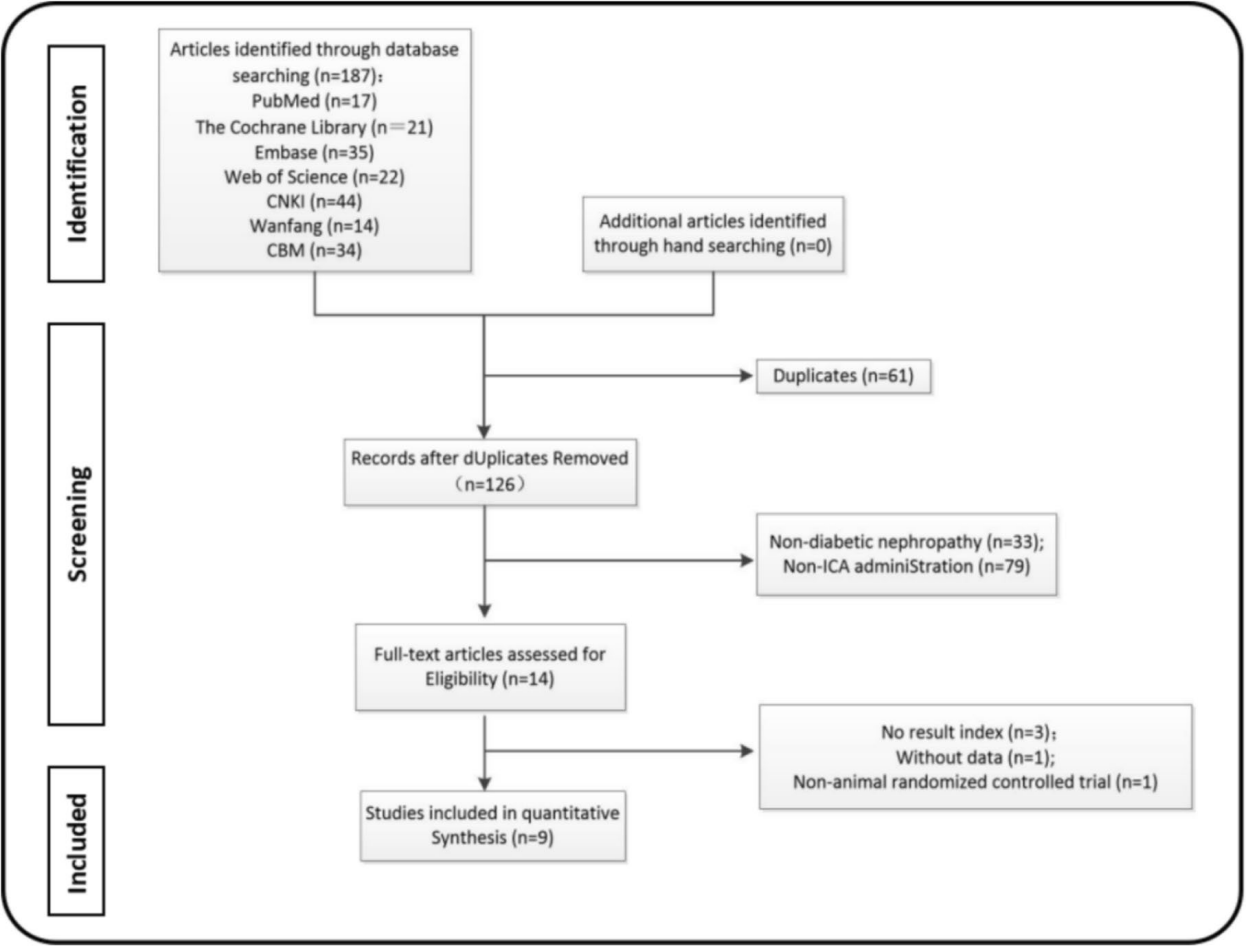
Flow chart of selecting process

**Fig. 3:**
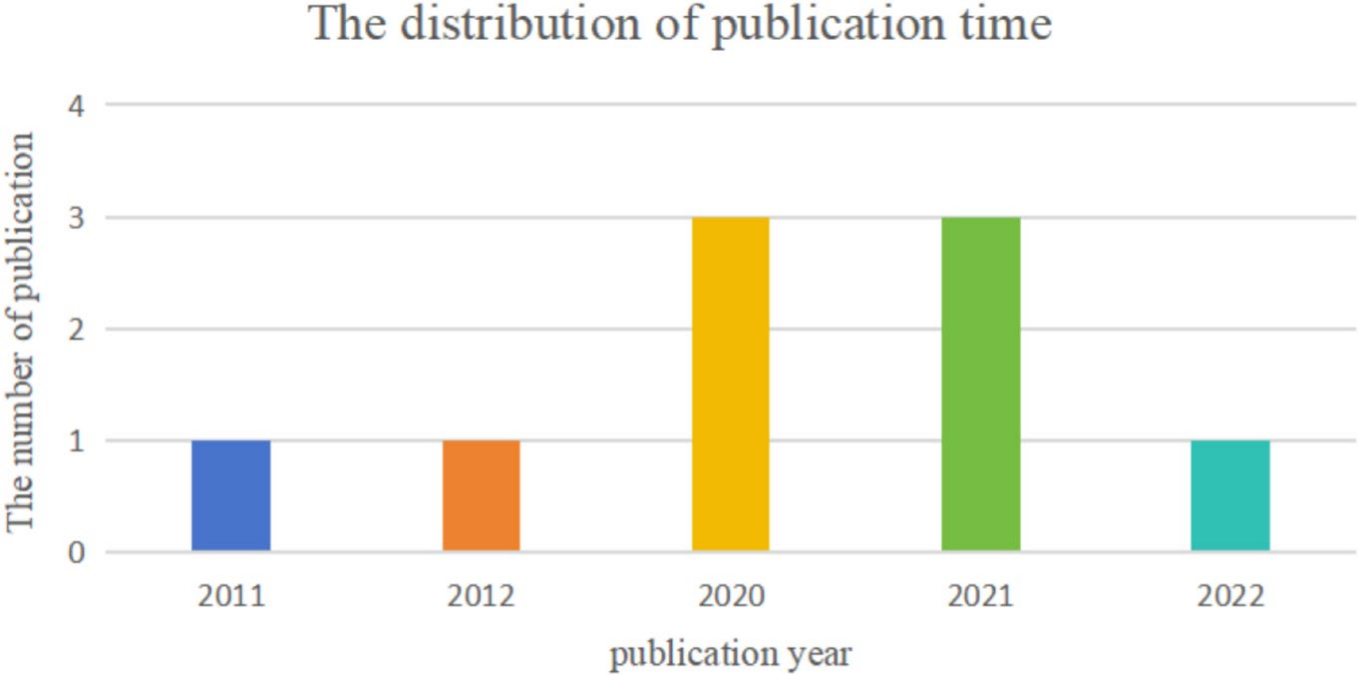
Publication year of the included studies

The methodology for literature assessment is depicted in Fig. 2, involving the acquisition of 187 articles from seven distinct databases, which were subsequently integrated into the Endnote platform. From this initial pool, 61 duplicates were meticulously eliminated. Subsequently, a dual-author, independent review process targeted titles and/or abstracts, resulting in the exclusion of 112 articles deemed irrelevant to the study. A more thorough examination of the full texts led to the further elimination of five articles, ultimately yielding a selection of 9 articles for inclusion [20–23, 26–30]. Notably, all included studies span the last eleven years (2011–2022; Fig. 3), underscoring the heightened research interest in the protective role of icariin against DN within the scientific community over the recent decade.

### Characteristics of included studies

**Fig. 4:**
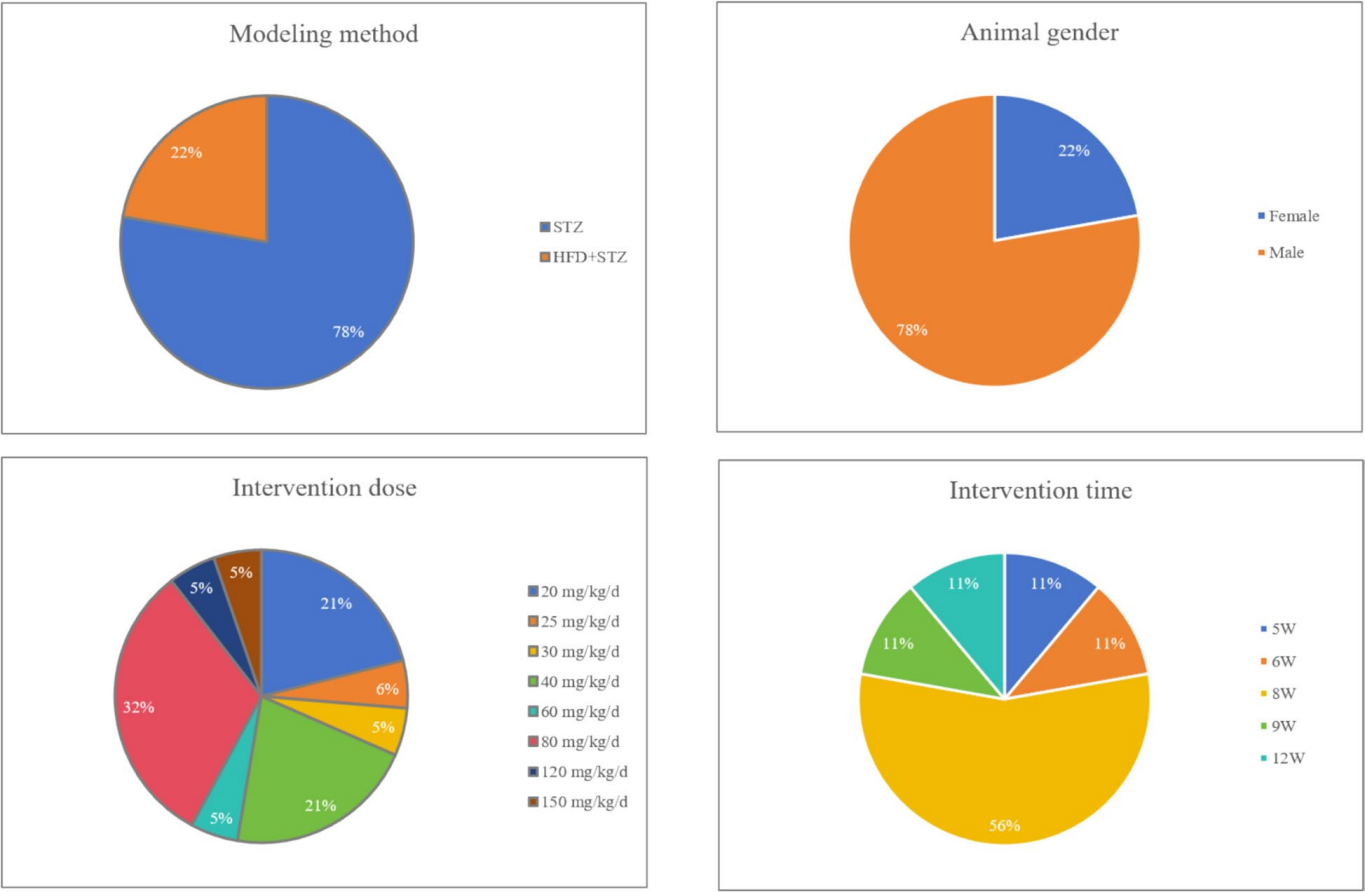
Characteristics of included studies

The following nine studies, comprising three in Chinese and six in English, were analyzed. Their key attributes are summarized as: (1) 308 diabetic model animals participated in the study, including treatment group (n = 154) and model group (n = 154). Eight studies employed Sprague Dawley rats [21–23, 26–30], while one study utilized C57BL/6 J mice [20]. (2) Seven studies focused on male animals [20, 22, 23, 26, 28–30], and two on females [21, 27]. (3) Regarding animal baseline characteristics, seven studies reported body weights [21–23, 26–28, 30], and five reported ages [22, 26–28, 30]. (4) Animal grouping varied, with three studies using ten animals per group [21, 27, 28], four using eight [20, 22, 26, 29], and two using six animals per group [23, 30]. (5) Diabetes induction methods differed, with seven studies opting for a simple intraperitoneal injection of STZ [20, 21, 23, 26, 27, 29, 30], while two studies combined a high-fat diet with STZ injection [22, 28]. (6) Eight studies administered the intervention orally [20, 22, 23, 26–30], with one study not specifying the route [21]. Doses ranged from 20 to 150 mg/kg/day, over a period of 5 to 9 weeks. (7) Outcome measures varied, with blood glucose recorded in six studies, BUN and SCR in all nine, 24 h UP in four, KI in four, 24 h urine output in three, MDA and SOD in five, GPX in two, TG and TC in two, and IL-1β in two studies. The characteristics of inclusion in the study are shown in Fig. 4 and Table 1.

**Table 1:** Characteristics of included studies

Study	Country	Species,Sex, weight, Age	Sample sizes(treatment/control)	Modeling method and standard	Icariin intervention (administration, drug dose, duration)	Outcomes
Chen 2012	China	SD Rats, male, 180–200 g, 8 weeks	8/8	Caudal vein injection of STZ(40 mg/kg), BG > 16.7 mmol/L	By Intragastric, 80 mg/kg/d, 8w	FBG、SCR、BUN、MDA、SOD
Cheng 2020	China	SD Rats, Female, 180–200 g, 8 weeks	10/10	Intraperitoneal injection of STZ(55 mg/kg), FBG > 16.7 mmol/L	By Intragastric, 30、60、120 mg/kg/d, 8w	SCR、BUN、TG、TC
Ding 2022	China	SD Rats, male, 160–180 g, ?	6/6	Intraperitoneal injection of STZ(55 mg/kg), BG > 16.7 mmol/L	20、40、80 mg/kg/d 8w	FBG、RBG、SCR、BUN、24 h UP、IL1β
Jia 2021	China	SD Rats, male, 160–180 g, 6 weeks	8/8	HFD + Intraperitoneal injection of STZ(35 mg/kg), FBG > 11.1 mmol/L RBG > 16.7 mmol/L	By Intragastric, 20、40、80 mg/kg/d, 12w	SCR、BUN、24 h UP
Qi 2011	China	SD Rats, male, ? ?	8/8	Caudal vein injection of STZ(40 mg/kg), FBG > 16.7 mmol/L	By Intragastric, 80 mg/kg/d, 8w	RBG、SCR、BUN、MDA、SOD
Qi 2021	China	ICR mice, male, ?, ?	8/8	Intraperitoneal injection of STZ(150 mg/kg), FBG ≥ 16.7 mmol L	By Intragastric, 150 mg/kg/d, 6w	FBG、SCR、BUN、MDA、SOD、24 h UP
Wang 2020	China	SD Rats, female, 180–200 g, ?	10/10	Intraperitoneal injection of STZ(55 mg/kg), FBG > 11.1 mmol/L RBG > 16.7 mmol/L	By Intragastric, 20、40、80 mg/kg/d, 9w	FBG、RBG、SCR、BUN、MDA、SOD、24 h UP、TG、TC
Zang 2021	China	SD Rats, male, 200–320 g, 6–8 weeks	6/6	Intraperitoneal injection of STZ(55 mg/kg) FBG > 16.7 mmol/L	By Intragastric, 20、40、80 mg/kg/d, 5w	SCR、BUN
Zhao 2020	China	SD Rats, male, 180–220 g, 4–6 weeks	10/10	HFD + Intraperitoneal injection of STZ(60 mg/kg), FBG > 16.7 mmol/L	By Intragastric, 25 mg/kg/d, 8w	SCr、BUN、MDA、SOD、IL-1β

The evaluation of the methodological rigor across the reviewed studies was undertaken utilizing the SYRCLE risk bias instrument, as summarized in Table 2. Notably, a substantial deficit in fulfilling comprehensive analytical standards emerged, primarily stemming from inadequate reporting practices. While all studies affirmed the random assignment of animal groups, crucial details such as the methodology for generating random sequences and concealment of allocation were conspicuously absent. Six studies (66.7%) did not report random feeding information, and the risk of bias was not clear. Additionally, three studies (23.5%) encountered data gaps, lacking explanations on the implications of these omissions on the validity of final findings, heightening concerns about attrition bias. Nine studies reported all expected results, and we believe that the risk of bias in selective results reports is low. According to our judgment, there are no other biased problems in all the literature. In addition, the risk of bias in the following areas is not described: baseline characteristics, blind approaches for ‘‘researchers’’ and/or ‘‘outcome evaluators’’, and random outcome assessments. Neither of the two independent authors found any other deviations.

**Table 2:** Risk of bias of included studies

Study	Random Sequence Generation	Baseline Characteristics	Allocation concealment	Random Housing	blinding (caregivers and/or researchers)	Random outcome assessment	blinding (outcome assessor)	Incomplete Outcome Data	Selective outcome reporting	Other Sources of Bias	Overall risk
Chen 2012	Unclear	Unclear	Unclear	Low risk	Unclear	Unclear	Unclear	High risk	Low risk	Low risk	High risk
Cheng 2020	Unclear	Unclear	Unclear	Low risk	Unclear	Unclear	Unclear	Low risk	Low risk	Low risk	High risk
Ding 2022	Unclear	Unclear	Unclear	Unclear	Unclear	Unclear	Unclear	High risk	Low risk	Low risk	High risk
Jia 2021	Unclear	Unclear	Unclear	Unclear	Unclear	Unclear	Unclear	Low risk	Low risk	Low risk	High risk
Qi 2011	Unclear	Unclear	Unclear	Unclear	Unclear	Unclear	Unclear	unclear	Low risk	Low risk	High risk
Qi 2021	Unclear	Unclear	Unclear	Unclear	Unclear	Unclear	Unclear	unclear	Low risk	Low risk	High risk
Wang 2020	Unclear	Unclear	Unclear	Unclear	Unclear	Unclear	Unclear	Low risk	Low risk	Low risk	High risk
Zang 2021	Unclear	Unclear	Unclear	Low risk	Unclear	Unclear	Unclear	High risk	Low risk	Low risk	High risk
Zhao 2020	Unclear	Unclear	Unclear	Unclear	Unclear	Unclear	Unclear	Low risk	Low risk	Low risk	High risk
Chen 2012	Unclear	Unclear	Unclear	Low risk	Unclear	Unclear	Unclear	High risk	Low risk	Low risk	High risk
Cheng 2020	Unclear	Unclear	Unclear	Low risk	Unclear	Unclear	Unclear	Low risk	Low risk	Low risk	High risk
Ding 2022	Unclear	Unclear	Unclear	Unclear	Unclear	Unclear	Unclear	High risk	Low risk	Low risk	High risk
Jia 2021	Unclear	Unclear	Unclear	Unclear	Unclear	Unclear	Unclear	Low risk	Low risk	Low risk	High risk

## Effectiveness

### Primary outcomes

#### Effect of ICA on blood glucose

**Fig. 5:**
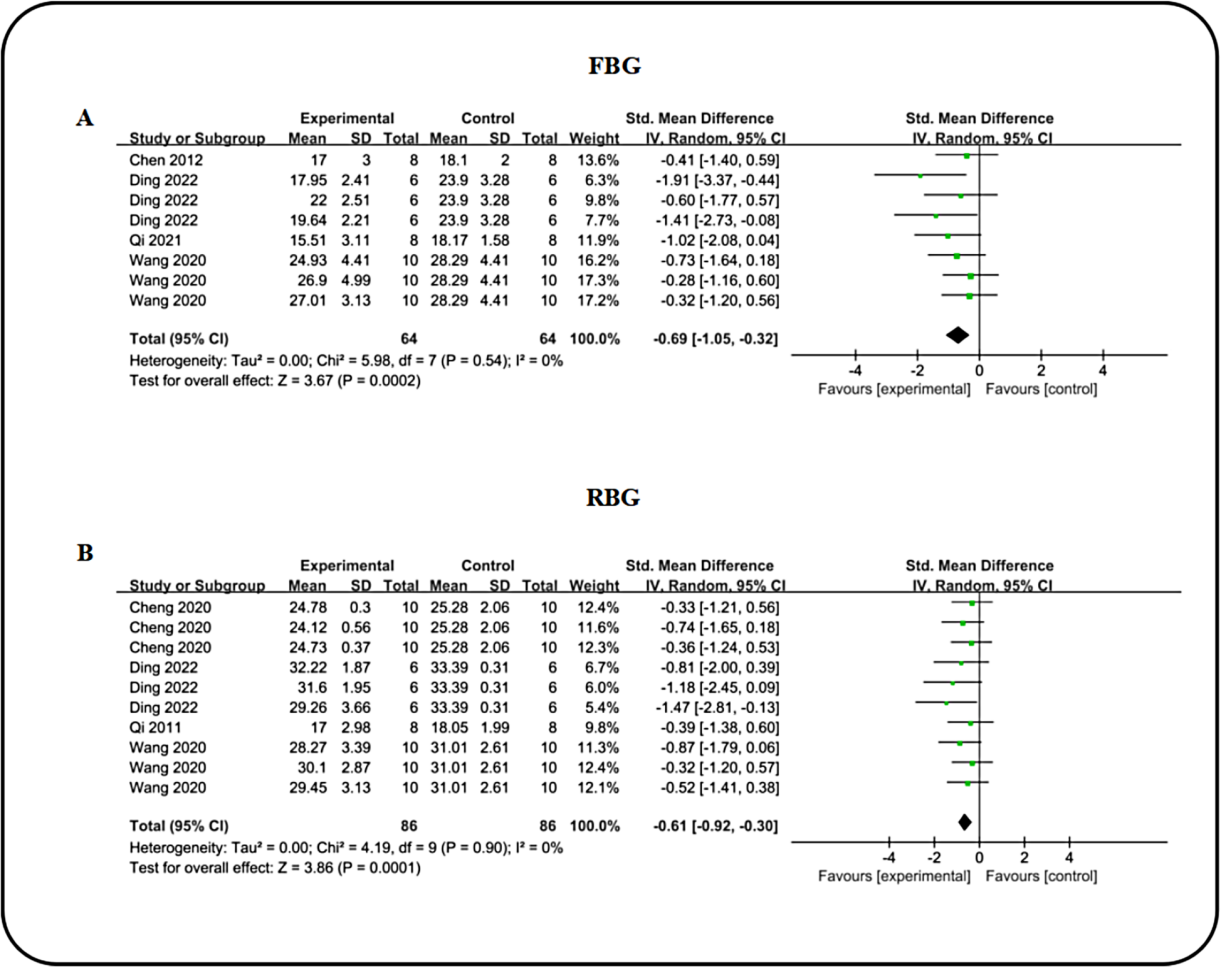
Forest plot: effect of ICA on FBG **A** and RBG **B** level

Four studies contributed data pertaining to blood glucose levels[including random blood glucose (RBG) and fasting blood glucose (FBG)], with comprehensive analysis revealing that icariin significantly decreased both FBG [n = 128, SMD: −0.69 (95% CI  1.05, − 0.32), P = 0.0002; I^2^ = 0%, P = 0.54; Fig. 5A] and RBG [n = 172, SMD: − 0.61 (95% CI: − 0.92, − 0.30), P = 0.0001; I^2^ = 0%, P = 0.9; Fig. 5B] compared to the DN group. Additionally, we compared the efficacy of ICA and ARB in regulating blood glucose. Out of the three studies reporting on FBG and two on RBG, the consolidated results indicated no statistically significant difference in the magnitude of FBG or RBG alterations between ICA and ARB treatments (Table 3).

**Table 3:** Summary of the outcome comparing with RAS inhibitor meta-analysis results

						Heterogeneity		
Outcome	No.of studies	Sample	SMD	95%CI	P	X^2^	I^2^ %	P
RBG	2	96	0.21	− 0.29 to 0.72	0.41	7.31	32	0.20
FBG	3	112	− 0.01	− 0.39 to 0.36	0.95	4.13	0	0.66
SCR	4	132	0.11	− 0.51 to 0.73	0.73	20.55	66	0.004
BUN	4	132	0.35	− 0.49 to 1.18	0.41	32.44	78	< 0.0001
24 h UP	3	112	0.09	− 0.97 to 1.15	0.86	33.81	82	< 0.00001
24 h UV	3	112	0.59	− 0.05 to 1.22	0.07	14.88	60	0.02
KI	3	112	− 0.04	− 0.72 to 0.64	0.91	17.34	65	0.008
SOD	3	96	− 0.70	− 2.30 to 0.90	0.39	42.41	91	< 0.00001
MDA	3	96	0.41	− 0.33 to 1.15	0.28	12.30	67	0.02
GPX	2	36	0.58	− 0.20 to 1.35	0.15	1.31	24	0.25
IL-1β	2	56	0.37	− 1.15 to 1.88	0.64	17.16	83	0.0007

### Effect of ICA on renal function index

**Fig. 6:**
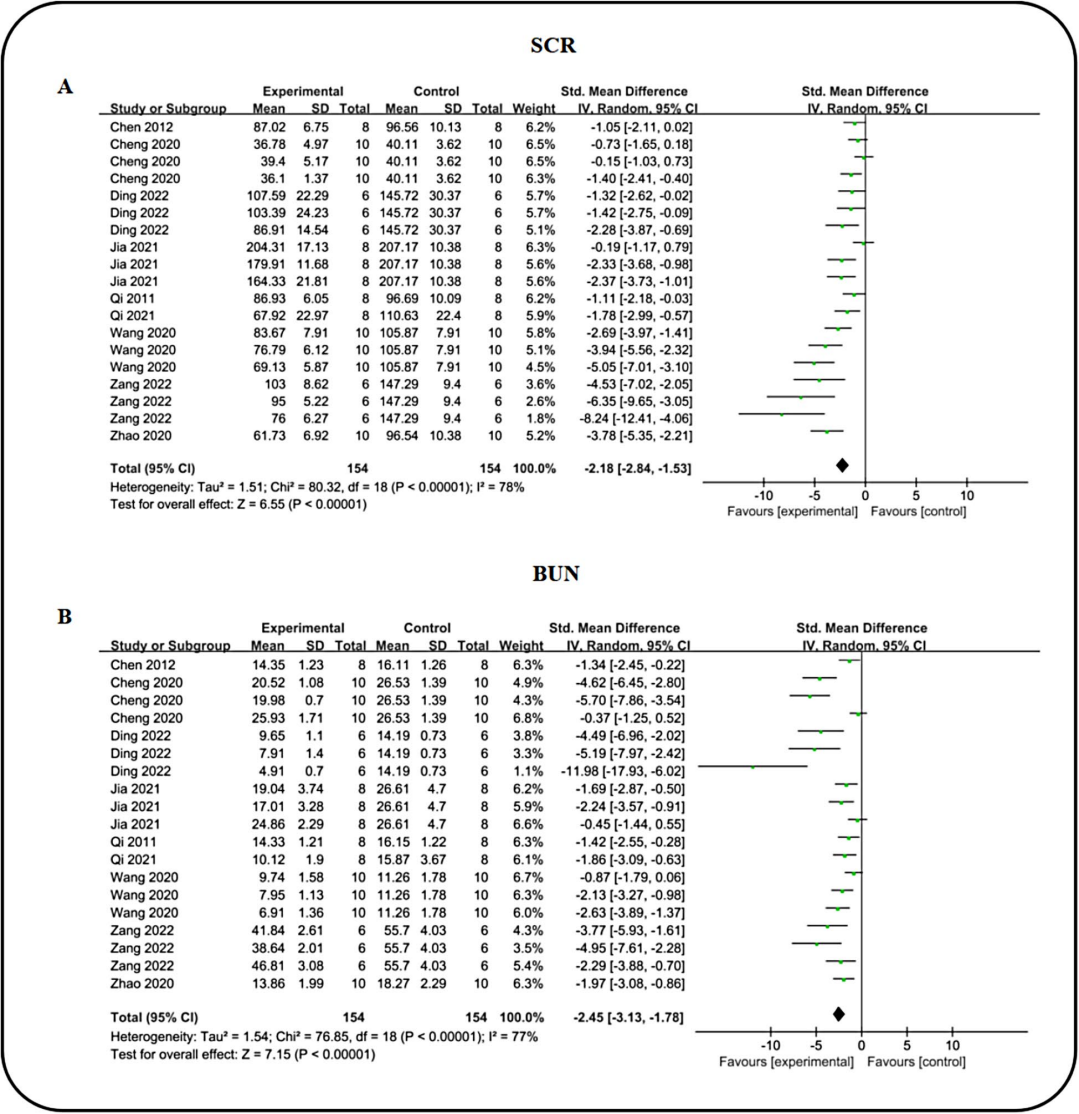
Forest plot: effect of ICA on SCR **A** and BUN **B** level

**Fig. 7:**
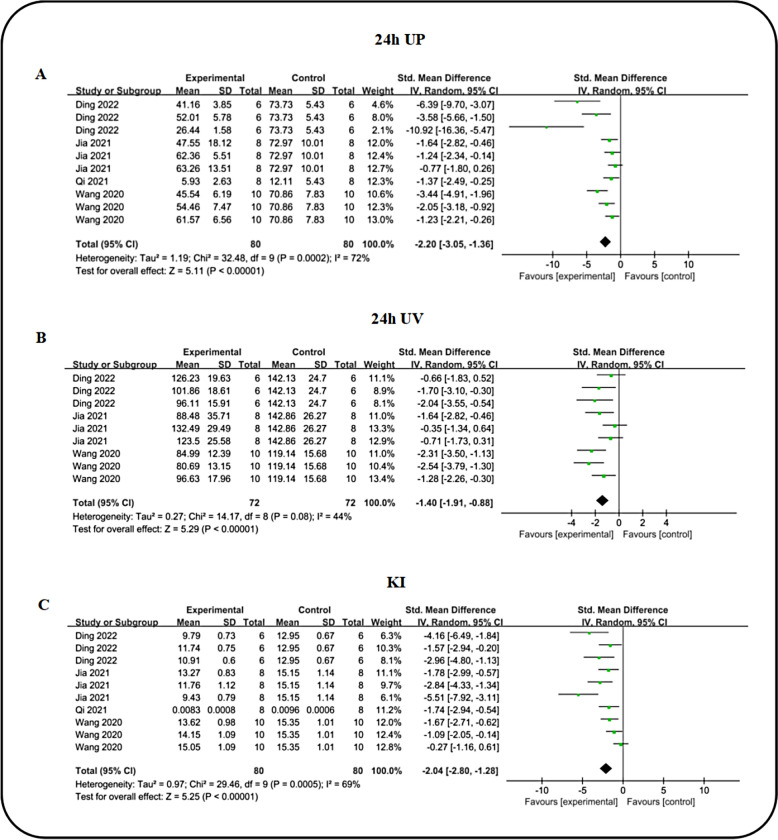
Forest plot: effect of ICA on 24 h UP **A**, 24 h UV **B** and KI **C** level

To assess renal function, we assessed key indicators including 24 h urinary protein excretion (24 h UP), serum creatinine (SCR), blood urea nitrogen (BUN), 24-h urinary volume (24 h UV), and kidney index (KI). Across nine studies examining SCR levels, meta-analysis demonstrated that icariin significantly lowered SCR [n = 308, SMD: − 2.18 (95% CI 2.84,  − 1.53), *P* < 0.00001; I^2^ = 78%, *P* < 0.00001; Fig. 6A] compared to the DN group. Concurrently, the collective findings from nine studies indicated that ICA favorably reduced BUN [n = 308, SMD: − 2.45 (95% CI − 3.13, − 1.78), *P* < 0.00001; I^2^ = 77%, *P* < 0.00001; Fig. 6B) levels. Four studies reported on 24 h UP changes, revealing that ICA effectively decreased 24 h UP (n = 160, SMD: − 2.20 (95% CI − 3.05, − 1.36), *P* < 0.00001; I^2^ = 72%, *P* = 0.0002; Fig. 7A) when compared to the DN group. Utilizing data from three studies, we analyzed 24 h UV volume and found a significant reduction in the ICA intervention group (n = 144, SMD: − 1.40 (95% CI: − 1.91, − 0.88), *P* < 0.00001; I^2^ = 44%, *P* = 0.08; Fig. 7B). Given that an elevated kidney-to-body weight ratio indicates renal swelling and damage, we assessed the impact of ICA on KI. Four studies reported on KI, consistently showing that ICA markedly reduced KI [n = 160, SMD: − 2.04 (95% CI − 2.80, − 1.28), *P* < 0.00001; I^2^ = 69%, *P* = 0.0005; Fig. 7C) in diabetic model animals compared to the DN group, suggesting significant improvement in renal function. Furthermore, a comparative analysis between ICA and ARB in enhancing renal function indices was conducted, involving 3 studies on 24 h UP, 4 on SCR, 4 on 24 h UV and 3 on KI. The results revealed no statistically significant difference in renal function improvement between ICA and ARB (Table 3).

### Secondary outcomes

#### Oxidative stress in renal tissues

**Fig. 8:**
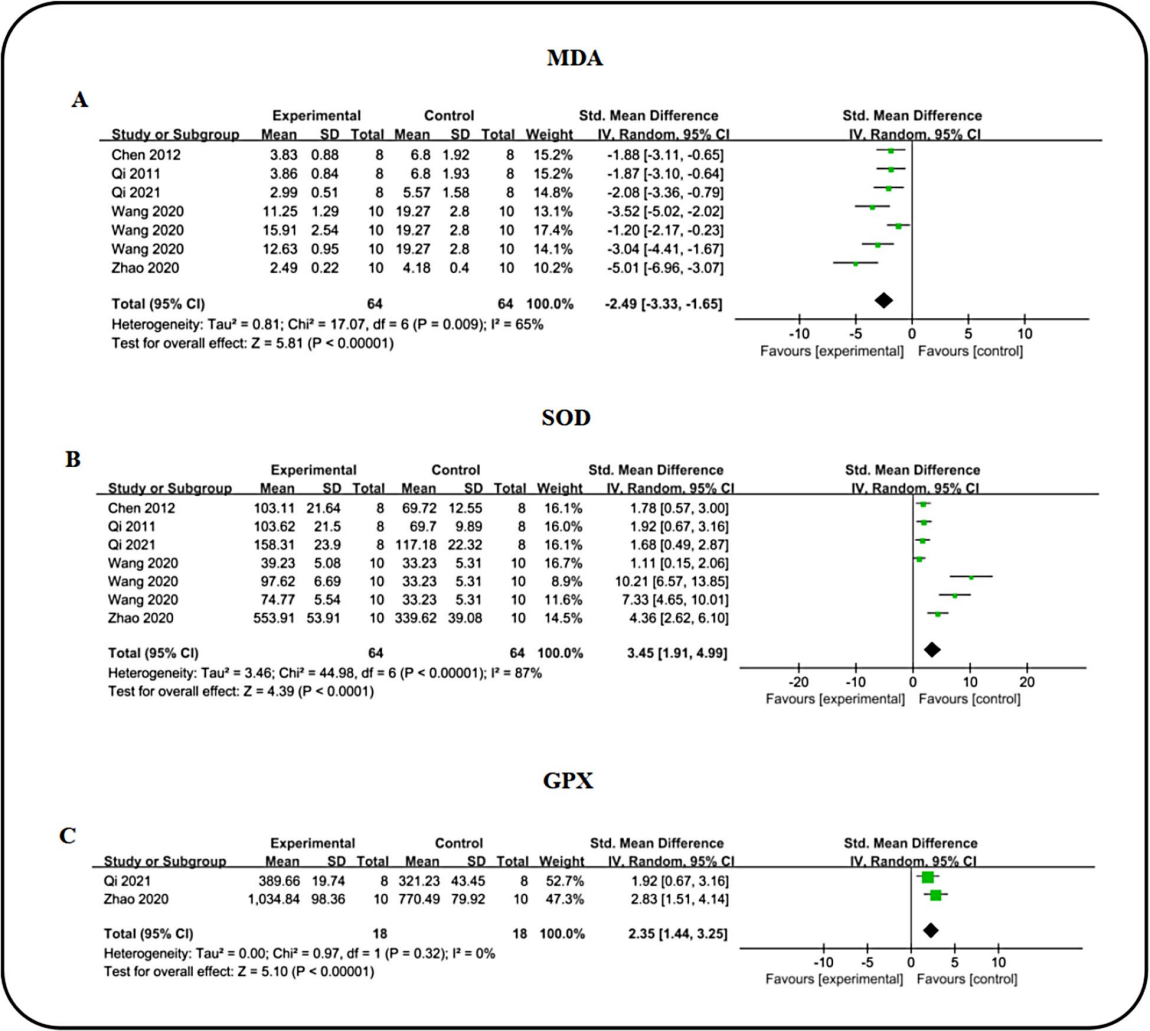
Forest plot: effect of ICA on MDA **A**, SOD **B** and GPX **C** level

In the context of hyperglycemia, mitochondria generate excessive reactive oxygen species (ROS), contributing to oxidative stress and renal injury. Utilizing malondialdehyde (MDA), Superoxide Dismutase (SOD) and Glutathione peroxidase (GPX) as outcome measures. To investigate the antioxidant effects of icariin on renal tissue, we analyzed five studies reporting MDA levels during DN treatment. The collective analysis demonstrated a notable anti-MDA (n = 128, SMD: − 2.49 (95% CI − 3.33, − 1.65), *P* < 0.00001; I^2^ = 65%, *P* = 0.009; Fig. 8A) effect of ICA. Similarly, assessment of SOD (n = 128, SMD: 3.45 (95% CI 1.91, 4.99), *P* < 0.0001; I^2^ = 87%, *P* < 0.00001; Fig. 8B) levels from five studies revealed a significant elevation in the ICA group compared to DN controls. Two studies measured GPX levels, showing that ICA intervention significantly enhanced GPX [n = 36, SMD: 2.35 (95% CI: 1.44, 3.25), *P* < 0.00001; I^2^ = 0%, *P* = 0.32; Fig. 8C) levels compared to the DN group. Overall, these findings indicate that ICA effectively upregulates antioxidant defenses and mitigates oxidative stress in renal tissue of DN animal models. Moreover, a comparative analysis between ICA and ARB in modulating oxidative stress indices was conducted, including 3 studies on SOD, 3 studies on MDA and 2 studies on GPX. The results revealed no statistically significant difference in renal function improvement between ICA and ARB (Table 3).

### Inflammatory biomarkers

**Fig. 9:**
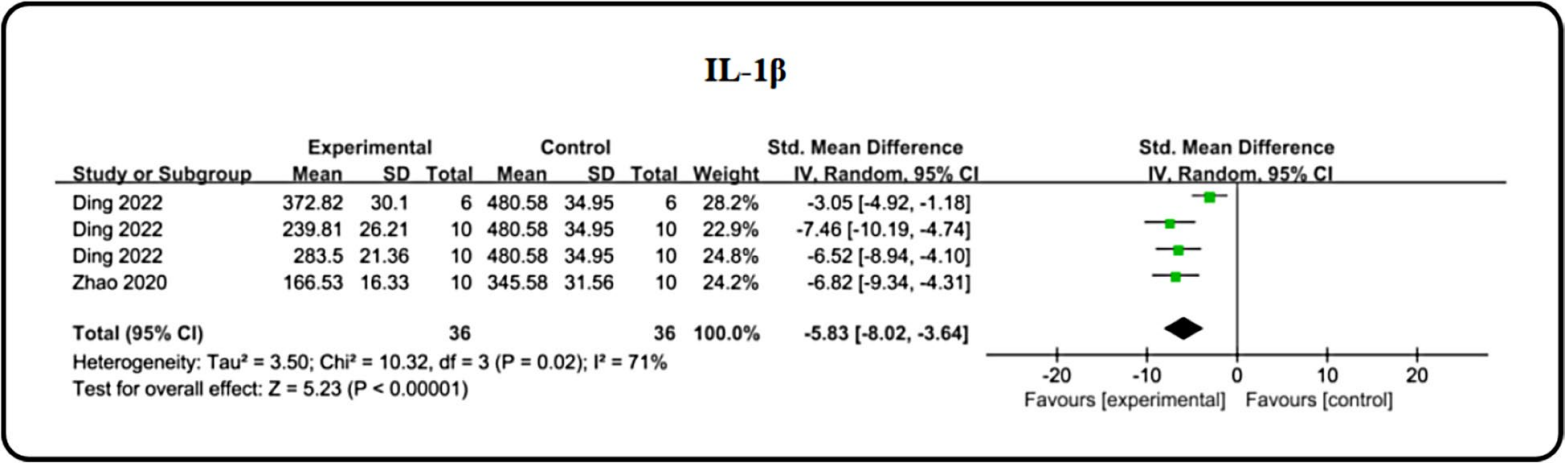
Forest plot: effect of exosome on IL-1β level

In hyperglycemic conditions, the liberation of inflammatory cytokines from immune cells triggers activation of signaling cascades such as NF-κB and NLRP3, ultimately leading to kidney damage. To assess the anti-inflammatory effects of icariin, we focused on key inflammatory biomarkers. Specifically, interleukin 1β (IL-1β) was employed as an outcome measure in two studies. A meta-analysis of these studies revealed a marked reduction in IL-1β [n = 72, SMD: − 5.83 (95%CI − 8.02, − 3.64), *P* < 0.00001; I^2^ = 71%, *P* = 0.02; Fig. 9] levels with ICA treatment compared to the DN model group. Additionally, we compared the anti-inflammatory potential of ICA and ARB using IL-1β as a metric across two studies. The findings indicated no statistically significant difference in the modulation of inflammatory factors between ICA and ARB (Table 3). However, it is noteworthy that data limitations have impacted the robustness and generalizability of these conclusions.

### Effects on lipid metabolism

**Fig. 10:**
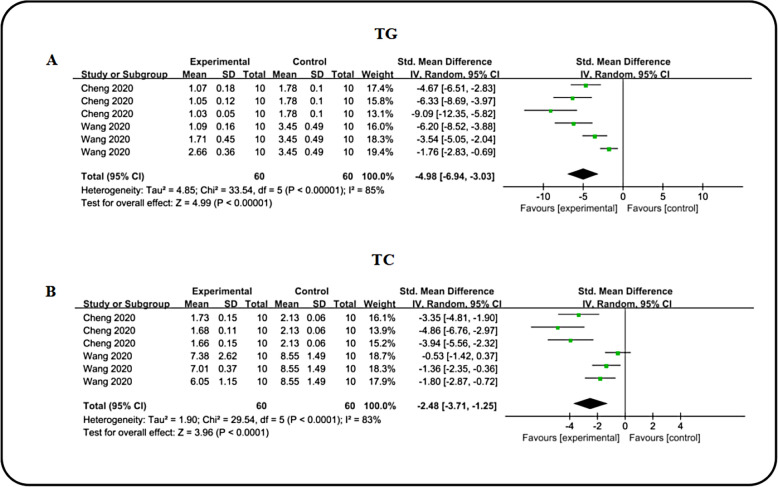
Forest plot: effect of ICA on TG **A** and TC **C** level

Diabetes frequently coexists with lipid metabolic disturbances, prompting a comprehensive evaluation of icariin's impact on lipid profiles. Utilizing, triglyceride (TG) and total Cholesterol (TC) as outcome measures in two studies, our consolidated analysis demonstrates that ICA effectively lowers TG [n = 120, SMD: − 4.98 (95% CI − 6.94, − 3.03), *P* < 0.00001; I^2^ = 85%, *P* < 0.00001; Fig. 10.A] and TC [n = 120, SMD: − 2.48 (95%CI− 3.71, − 1.25), *P* < 0.0001; I^2^ = 83%, *P* < 0.0001; Fig. 10B) levels in DN models. In essence, ICA contributes to the amelioration of lipid metabolism in DN.

### Kidney protective mechanisms

**Fig. 11:**
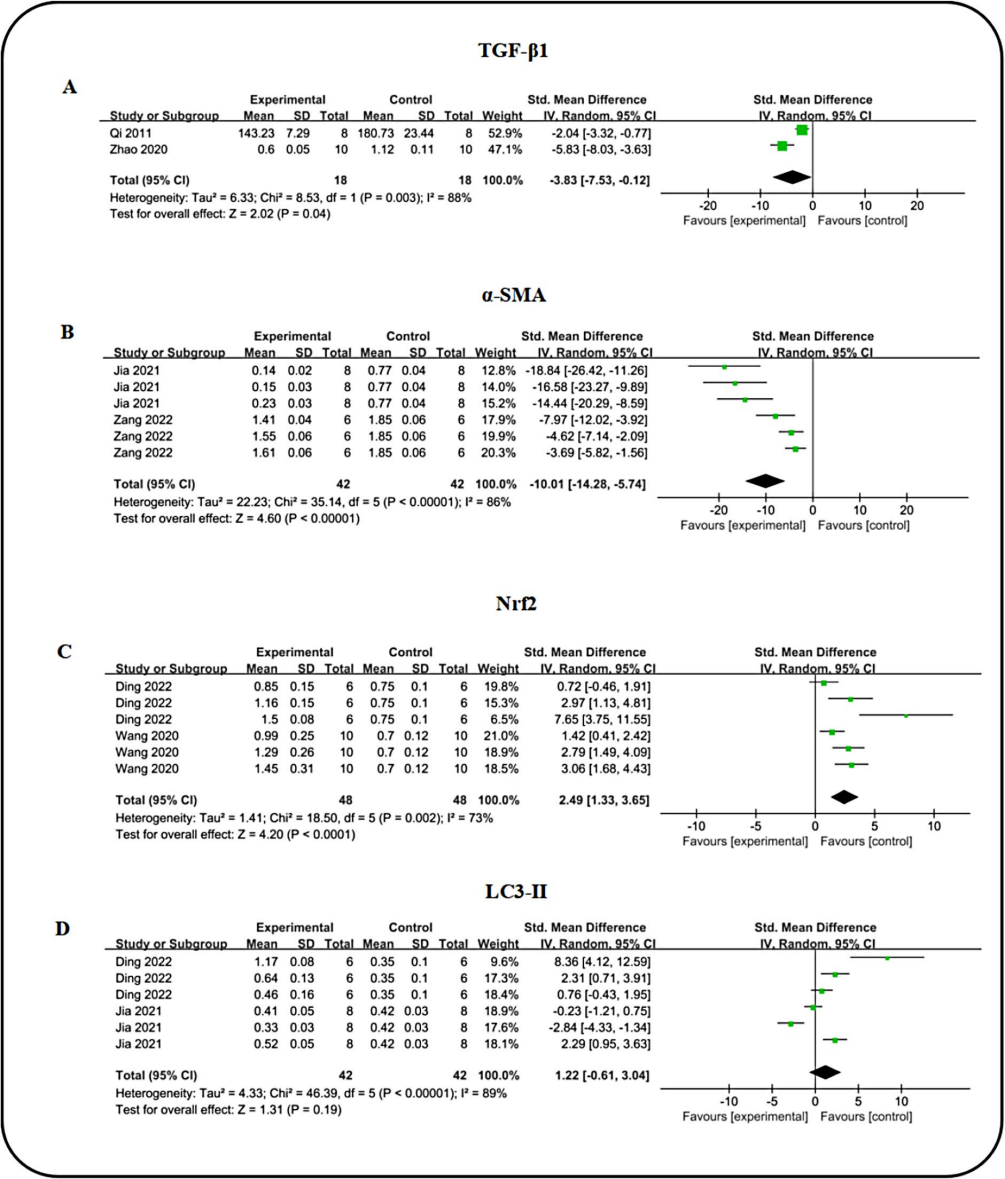
Forest plot: effect of ICA on TGF-β1 **A**, α-SMA **B**, Nrf2 **C** and LC3-II **D** level

Two studies reported on the expression of TGF-β1, a-SMA, Nrf2, and LC3-II. Comprehensive analysis revealed that ICA significantly decreased TGF-β (n = 36, SMD: − 3.83 (95%CI: − 7.53, − 0.12), *P* = 0.04; I^2^ = 88%, *P* = 0.003; Fig. 11A) and α-SMA (n = 84, SMD: − 10.01 (95%CI: − 14.28, − 5.74), *P* < 0.00001; I^2^ = 86%, *P* < 0.00001; Fig. 11B) expression while enhancing Nrf2 [n = 96, SMD: 2.49 (95%CI: 1.33, 3.65), *P* < 0.0001; I^2^ = 73%, *P* = 0.002; Fig. 11C) and LC3-II [n = 84, SMD: 1.22 (95%CI − 0.61, 3.04), *P* = 0.19; I^2^ = 89%, *P* < 0.00001; Fig. 11D] levels compared to the DN group. While additional proteins regulated by ICA were noted in individual studies, meta-analysis was limited by the small sample size (fewer than two reports). Nonetheless, these findings suggest a favorable role for ICA in the anti-renal injury mechanism, with a statistical significance (*P* < 0.5).

### Sensitivity analysis

A sensitivity analysis was conducted across all outcome indicators. Notably, upon excluding the study by Ding et al. [23], the heterogeneity associated with 24 h UP significantly diminished from 72 to 40%. This study had a notable influence on the overall heterogeneity of the results, yet icariin consistently demonstrated a significant reduction in 24 h UP [SMD = − 2.20 (− 3.05, − 1.36), *P* < 0.00001; SMD = − 1.57 (− 2.13, − 1.02), *P* < 0.00001; Table 4. Sensitivity analysis for 24 h UP] levels, both with and without the inclusion of the Ding et al. study. Upon excluding the study by Zhao et al. [28], the heterogeneity in MDA levels notably decreased from 65 to 43%. This suggests that the Zhao et al. study may contribute to the heterogeneity observed in 24 h UP outcomes. Icariin consistently demonstrated a significant reduction in MDA [SMD = − 2.49 (− 3.33, − 1.65), *P* < 0.00001; SMD = −2.16 (− 2.83, − 1.48), *P* < 0.00001; Table 5] levels, both with and without the inclusion of the Zhao et al. study. The sensitivity analysis did not significantly alter the meta-analysis results for other outcome measures, and the sources of heterogeneity remain unclear for these parameters.

**Table 4:** Sensitivity analysis for 24 h UP

	SMD	p	I^2^ %
With	− 2.20 [− 3.05, − 1.36]	*P* < 0.00001	72
Without	− 1.57 [− 2.13, − 1.02]	*P* < 0.00001	40

**Table 5:** Sensitivity analysis for MDA

	SMD	p	I^2^ %
With	− 2.49 [− 3.33, − 1.65]	*P* < 0.00001	65
Without	− 2.16 [− 2.83, − 1.48]	*P* < 0.00001	43

### Subgroup analysis

Given the substantial heterogeneity across studies, the primary findings were further explored through subgroup analyses stratified by icariin dosage and treatment duration. This analysis encompassed SCR, BUN, 24 h UP, KI, 24 h UV, and FBG, while other outcomes were excluded due to insufficient study numbers.

### Grouped according to ICA dose

Icariin doses were categorized into three subgroups: ≤ 30 mg/kg, 30–80 mg/kg, and ≥ 80 mg/kg. The summary results of 9 studies showed that the level of SCR in each subgroup decreased significantly after icariin treatment with significant heterogeneity (SMD = − 1.98 (− 3.20, − 0.77), *P* = 0.001, I^2^ = 81%; SMD = − 2.45 (− 4.14, − 0.76), *P* = 0.004, I^2^ = 85%; SMD = − 2.24 (− 3.16, − 1.31), *P* < 0.00001, I^2^ = 72%; Table 6), and there were significant differences among subgroups (*P* < 0.00001).Similarly, summary analyses of BUN levels in subgroups showed a reduction compared to the control group with significant heterogeneity [SMD = − 1.39 (− 2.26, − 0.51), *P* = 0.002, I^2^ = 69%; SMD = − 3.18 (− 4.47, − 1.88), *P* < 0.00001, I^2^ = 67%; SMD = − 2.90 (− 4.05, − 1.76), *P* < 0.00001, I^2^ = 76%; Table 6] and significant differences between subgroups (*P* < 0.00001). The results of four studies showed that compared with the DN model group, ICA decreased the level of 24 h UP in each subgroup with significant heterogeneity [SMD = − 1.54 (− 2.77, − 0.32), *P* = 0.01, I^2^ = 64%; SMD = − 2.52 (− 4.35, − 0.70), *P* = 0.007, I^2^ = 76%; SMD = − 2.87 (− 4.69, − 1.04), *P* = 0.002, I^2^ = 81%; Table 6], and there were significant differences among subgroups (*P* < 0.00001). The data obtained from 4 studies reported KI, and the summary results showed that after ICA treatment, each subgroup decreased significantly, with significant heterogeneity [SMD = − 1.12 (− 2.15, − 0.09), *P* = 0.03, I^2^ = 59%; SMD = − 2.14 (− 3.48, − 0.81), *P* = 0.002, I^2^ = 63%; SMD = − 2.93 (− 4.49, − 1.37), *P* = 0.0002, I^2^ = 74%; Table 6], and there were significant differences among subgroups (*P* < 0.00001). Meta-analysis showed that different doses of quercetin (≤ 30 mg/kg, 30–80 mg/kg and ≥ 80 mg/kg) could lower blood glucose, with higher doses exhibiting superior therapeutic outcomes. Based on these findings, we hypothesize that icariin doses exceeding 30 mg/kg may be more advantageous for the management of DN. In addition, subgroup analysis showed that ICA dose was not the source of heterogeneity among SCR, BUN, 24 h UP and KI studies.

**Table 6:** Subgroup analysis for renal function indices according to icariin doses

Subgroups/Outcomes	≤ 30 mg/kg			30–80 mg/kg			≥ 80 mg/kg			Subgroup difference
	SMD	P	I^2^ %	SMD	P	I^2^ %	SMD	P	I^2^ %	P
SCR	− 1.98 [− 3.20, − 0.77]	0.001	81	− 2.45 [− 4.14, − 0.76]	0.004	85	− 2.24 [− 3.16, − 1.31]	< 0.00001	72	< 0.00001
BUN	− 1.39 [− 2.26, − 0.51]	0.002	69	− 3.18 [− 4.47, − 1.88]	< 0.00001	67	− 2.90 [− 4.05, − 1.76]	< 0.00001	76	< 0.00001
24 h UP	− 1.54 [− 2.77, − 0.32]	0.01	64	− 2.52 [− 4.35, − 0.70]	0.007	76	− 2.87 [− 4.69, − 1.04]	0.002	81	< 0.00001
KI	− 1.12 [− 2.15, − 0.09]	0.03	59	− 2.14 [− 3.48, − 0.81]	0.002	63	− 2.93 [− 4.49, − 1.37]	0.0002	74	< 0.00001

### Grouped according to ICA treatment duration

ICA with different treatment duration was divided into two subgroups: ≤ 8 weeks and > 8 weeks. The summary results of 9 studies showed that the level of SCR in each subgroup decreased significantly after icariin treatment with significant heterogeneity [SMD = − 1.92 (− 2.65, − 1.19), *P* < 0.00001, I^2^ = 74%; SMD = −2.65 (− 4.00, − 1.30), *P* = 0.0001, I^2^ = 83%; Table 7], and there were significant differences among subgroups (*P* < 0.00001).Similarly, the summary analysis of BUN levels in subgroups showed a reduction compared to the control group with significant heterogeneity [SMD = − 3.10 (− 4.12, − 2.08), *P* < 0.00001, I^2^ = 81%; SMD = − 1.59 (− 2.29, − 0.89), *P* < 0.00001, I^2^ = 57%; Table 7], and there were significant differences among subgroups (*P* < 0.00001).The results of four studies showed that compared with the DN model group, ICA decreased the level of 24-h UP in each subgroup with significant heterogeneity [SMD = − 4.76 (− 7.88, − 1.63), *P* = 0.003, I^2^ = 85%; SMD = − 1.62 (− 2.28, − 0.97), *P* < 0.00001, I^2^ = 50%; Table 7], and there were significant differences among subgroups (*P* < 0.00001).The data obtained from 4 studies reported KI. The summary results showed that after ICA treatment, each subgroup of KI decreased significantly with significant heterogeneity [SMD = − 2.30 (− 3.30, − 1.29), *P* < 0.00001, I^2^ = 37%; SMD = − 1.88 (− 2.91, − 0.84), *P* = 0.0004, I^2^ = 77%; Table 7], and there were significant differences among subgroups (*P* < 0.00001). In summary, the duration of icariin treatment was not identified as a source of heterogeneity among the studies analyzed. Notably, treatment durations ranging from 5 to 8 weeks demonstrated relatively superior efficacy.

**Table 7:** Subgroup analysis for renal function indices according to icariin treatment duration

Subgroups/Outcomes	≤ 8 W			> 8 W			Subgroup difference
	SMD	P	I^2^^%^	SMD	P	I^2^ %	P
SCR	− 1.92[− 2.65,− 1.19]	< 0.00001	74	− 2.65[14.00, − 1.30]	0.0001	83	< 0.00001
BUN	− 3.10[− 4.12,− 2.08]	< 0.00001	81	− 1.59[− 2.29, − 0.89]	< 0.00001	57	< 0.00001
24 h UP	− 4.76[− 7.88,− 1.63]	0.003	85	− 1.62[− 2.28, − 0.97]	< 0.00001	50	< 0.00001
KI	− 2.30[− 3.30,− 1.29]	< 0.00001	37	− 1.88[− 2.91, − 0.84]	0.0004	77	< 0.00001

### Grouped according to animal model

We performed formal interaction tests to assess model differences across SCR, BUN, 24 h UP, and KI subgroups. The results demonstrated statistically significant differences between STZ and HFD/STZ models (interaction effects: SCR, *P* < 0.00001; BUN, *P* < 0.00001; 24 h UP, *P* < 0.00001; KI, *P* < 0.00001; Table 8S).

### Grouped according to animal gender

Formal interaction tests were conducted to assess sex differences in the SCR, BUN, 24 h UP, and KI subgroups. The results confirmed statistically significant differences between males and females (interaction effects: SCR, *P* < 0.00001; BUN, *P* < 0.00001; 24 h UP, *P* < 0.00001; KI, *P* < 0.00001; Table 9S).

### Publication bias

**Fig. 12:**
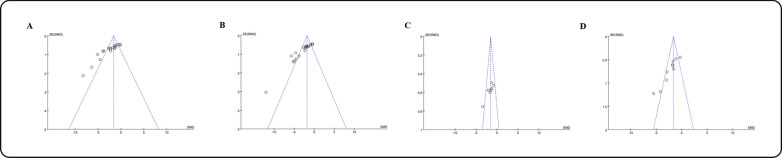
Funnel plots for SCR **A**, BUN **B**, 24 h UP **C** and KI **D** of publication bias

Publication bias can significantly influence the outcomes of meta-analyses. To assess this, we constructed funnel plots for SCR, BUN, 24 h UP, and KI, using SE as the measure. Our analysis indicated that there is publication bias in these results, the funnel diagram is not completely symmetrical, and the vast majority of studies are distributed at the top of the funnel chart and concentrated in the middle ( \* MERGEFORMAT Fig. 12).

## Discussion

### Effectiveness and summary of evidence

Our comprehensive preclinical systematic review and meta-analysis, encompassing 9 studies with 308 experimental animals, represents the first of its kind to assess the efficacy and potential mechanisms of icariin in treating diabetic secondary renal injury. Our findings underscore the promising role of icariin in the management of animal DN, where it demonstrated beneficial effects on renal function markers (SCR、BUN, 24 h UP, 24 h UV, and KI), inflammatory biomarkers (IL-1β), oxidative stress parameters (SOD,GPX, MDA), and risk factors (TG, TC). These protective effects are likely linked to ICA's antioxidant, anti-fibrotic, anti-apoptotic, and anti-inflammatory properties, thereby providing valuable insights for future human clinical trials. It is noteworthy that while the fasting FBG levels in the icariin group were observed to be lower compared to the control group, the animals remained in a hyperglycemic state. This suggests that the potential benefit of ICA in regulating blood glucose levels may be modest.

### Subgroup analysis

Our methodology adhered to GRADE recommendations by downgrading the certainty of evidence for studies with high risk of bias rather than excluding them [31], as certain clinically significant data may still provide valuable insights. Importantly, our primary conclusions remained robust despite these potential biases.

When exploring the sources of heterogeneity in our meta-analysis, we acknowledge the multitude of factors to consider, including animal species, modeling approaches, and intervention methods. Given that the majority of included studies utilized rats as the model organism and employed similar methods of diabetes induction via STZ intraperitoneal injection, we deemed animal species and modeling techniques as unlikely contributors to the observed heterogeneity. To further investigate, we conducted a subgroup analysis of 24 h UP, SCR, and BUN, focusing on icariin's dosage and treatment duration. By categorizing icariin doses into ≤ 30 mg/kg, 30–80 mg/kg, and ≥ 80 mg/kg subgroups, we aimed to pinpoint potential sources of heterogeneity. However, despite this detailed categorization, the heterogeneity remained significant, indicating that icariin dosage may not be a primary contributor to the observed variability. By focusing on the dose range of 20–150 mg/kg (a commonly used preclinical range), our study provides robust evidence supporting the therapeutic efficacy of ICA within this dosage window. We performed formal interaction tests for subgroup comparisons of SCR, BUN, 24 h UP, and KI. The findings demonstrate that ICA's intervention dosage significantly influences treatment outcomes, with 30–80 mg/kg ICA showing more beneficial effects on DN. However, current studies have not reported potential saturation effects of high-dose ICA, such as plateau effects. Therefore, future research should conduct dose-escalation studies (> 80 mg/kg) to investigate potential saturation phenomena.

Further subgroup analyses were conducted to delve into potential sources of heterogeneity, with icariin treatment duration serving as a basis for categorization into ≤ 8 weeks and > 8 weeks subgroups. Despite these efforts, the heterogeneity remained substantial, suggesting that treatment duration alone may not be the primary driver of the observed variability. Notably, while no significant reduction in heterogeneity was observed across all subgroups, there were notable decreases in the heterogeneity of specific outcomes, such as KI and 24 h UP, within specific treatment duration subgroups. Specifically, for KI in the ≤ 8 weeks subgroup, heterogeneity decreased from 69 to 37%, and for 24 h UP in the > 8 weeks subgroup, it decreased from 72 to 50%. These findings may be influenced by the relatively small sample sizes and warrant further validation through larger-scale experiments. Nonetheless, our subgroup analyses provide preliminary indications that icariin treatment duration may contribute, to some extent, to the heterogeneity observed in this study.

Although model variations exist, they reflect the clinical heterogeneity of DN pathogenesis (e.g., hyperglycemia vs. metabolic syndrome). The consistency of our primary findings across different subgroups supports the robustness of ICA's renoprotective effects, which appear independent of specific induction methods. In summary, the dynamic variability of DN models significantly influences interindividual differences in drug efficacy and disease adaptation of icariin by modulating its metabolic conversion efficiency, spatiotemporal distribution, and target interactions. The consistency of our primary findings across sex subgroups supports the robustness of ICA's renoprotective effects. The observed sex differences in DN progression may stem from hormonal, metabolic, and immunological factors, in addition to methodological variations.

### Sensitivity analysis

Although the random-effects model accounts for between-study variations, the high I^2^ value suggests that pooled estimates should be interpreted with caution, as they may represent an average of clinically or methodologically heterogeneous effects. The exclusion of Ding et al. and Zhao et al. reduced heterogeneity in certain outcomes, but the observed variability may still originate from multiple combined factors, including differences in animal species, dosing regimens, and study designs.

In conducting a sensitivity analysis, Ding's study emerged as a notable contributor to the observed heterogeneity in the meta-analysis of 24 h UP. Upon close examination and comparison with other studies included in this meta-analysis, we postulate that human error could potentially be the primary driver of this discrepancy in 24 h UP outcomes. Despite utilizing the same testing kit across all studies, the results reported by Ding et al. stand out as markedly different from the rest, suggesting a potential role for human factors in the observed heterogeneity.

Zhao et al.'s investigation highlights the presence of heterogeneity in the meta-analysis of MDA. Upon thorough review and comparison with other meta-analyses on MDA, we identified a key distinction: the dosing regimen. Specifically, Zhao et al. employed a low-dose intervention (25 mg/kg/day), whereas other studies utilized doses ranging from 40 to 150 mg/kg. This variation in dosing strategies provides a plausible explanation for the observed heterogeneity. Furthermore, our analysis revealed that higher doses of icariin exhibited more pronounced therapeutic effects, suggesting a potential dose–response relationship.

### Potential mechanism of action

**Fig. 13:**
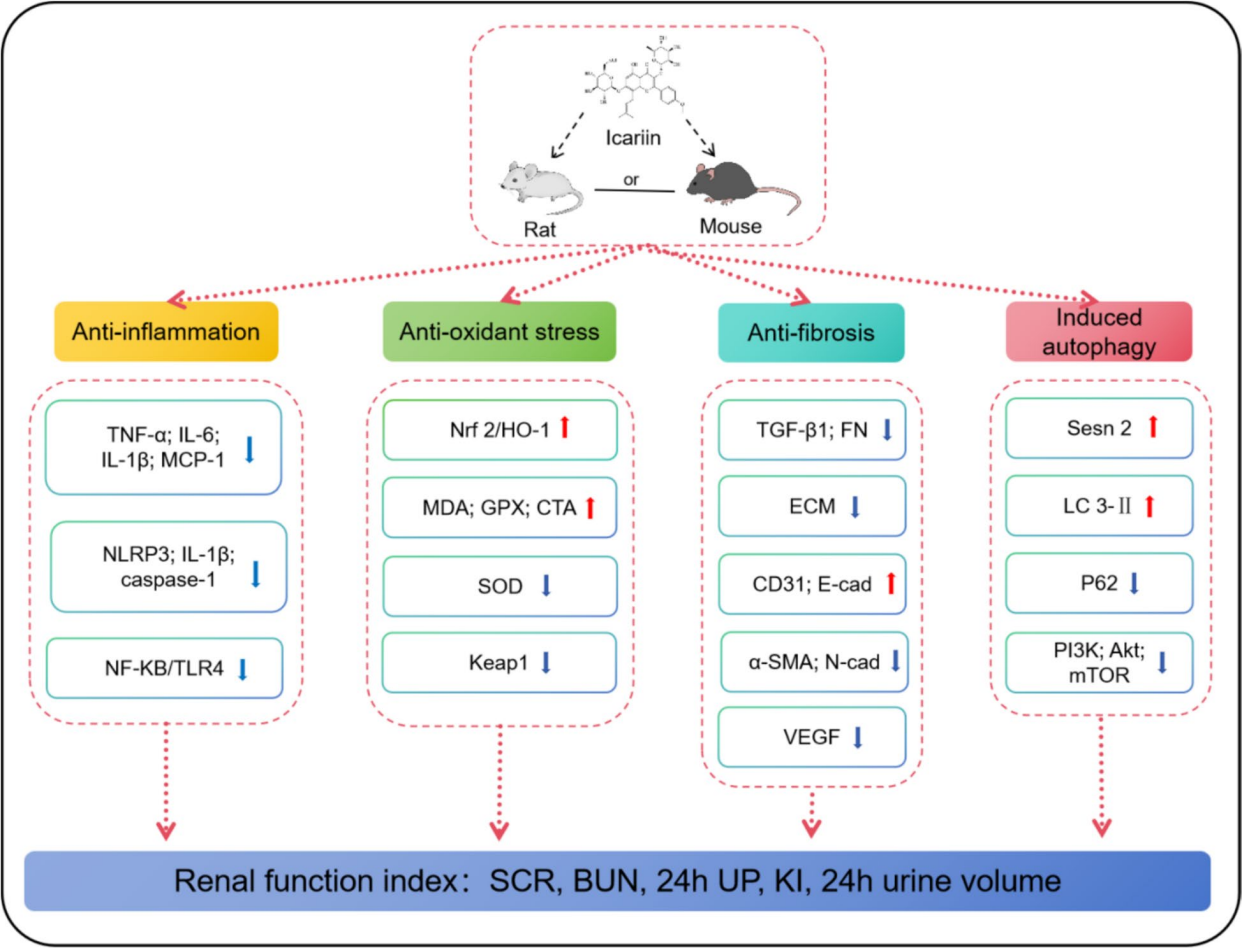
Mechanism of ICA in the treatment of Diabetic Nephropathy

The results of a systematic review of the included study showed that the main protective mechanisms of icariin included the following ( \* MERGEFORMAT Fig. 13): (1) The progression of DN towards ESRD is significantly influenced by renal interstitial fibrosis [32], a process that encompasses the activation of renal tubular epithelial cells, infiltration of inflammatory cells, release of fibrogenic factors, and the subsequent production of extracellular matrix (ECM) deposits [33]. Characteristic of diabetic nephropathy fibrosis is the excessive accumulation of type IV collagen [34], which is upregulated by TGF-β1 in all glomerular cells [35]. Icariin mitigates renal fibrosis-induced damage by inhibiting the overproduction of TGF-β1 and type IV collagen within the kidney [29]. Furthermore, renal EndMT, defined as the acquisition of mesenchymal traits by glomerular endothelial cells [36], is a pivotal driver of renal fibrosis. Icariin exerts its inhibitory effect on renal EndMT by modulating marker expression: enhancing the downregulation of endothelial markers (CD31, E-cad) and attenuating the upregulation of mesenchymal markers (α-SMA, N-cadherin, FN, FSP-1) [37]. Renal histological assessment revealed that icariin markedly mitigated diabetes-induced glomerular mesangial matrix expansion and reduced ECM accumulation in the mesangial region [29]. The underlying anti-fibrotic mechanism of icariin involves the activation of AR/RKIP, thereby inhibiting the MEK/ERK pathway and subsequent renal EndMT triggered by hyperglycemia [37]. (2) During the onset and progression of DN, hyperglycemia acts as a stimulus, enhancing NLRP-3 expression and triggering downstream inflammatory cascades [38]. Additionally, high glucose levels and angiotensin II (AngII) synergistically promote TLR4 expression and activation, which, in turn, stimulate downstream NFKB factors, leading to augmented production of proinflammatory cytokines and chemokines, including IL-6, MCP-1 and IL-1β [39, 40]. ICA exerts a protective effect against renal injury mediated by inflammatory factors like IL-1, IL-6, and TNF-α by suppressing NLRP3 inflammasomes and the NF-κB/TLR4 signaling axis [20, 23]. Despite indications of ICA's multifaceted protective roles, the precise molecular mechanisms and key pathways remain elusive. We propose that future investigations leverage high-throughput analytical approaches to elucidate ICA's potential as a primary target or a crucial pathway in the anti-DN therapeutic landscape. (3) Oxidative stress emerges as a pivotal pathogenic factor in DN, where hyperglycemia fosters excessive generation of ROS that directly harm renal tissues [41]. Concurrently, the body's innate antioxidant defenses are compromised, exacerbating oxidative stress in the context of DN [42]. SOD, the primary antioxidant enzyme, plays a vital role in scavenging free radicals, with its levels serving as a direct indicator of the body's antioxidant capacity. By eliminating superoxide free radicals, SOD mitigates kidney damage stemming from ROS [43]. Additionally, GPX, a crucial peroxidase, decomposes peroxides into harmless hydroxyl compounds, thereby safeguarding against oxidative insults [44]. MDA, the end product of lipid peroxidation arising from the attack of reactive oxygen species on polyunsaturated fatty acids in biofilms [45], serves as an indicator of the extent of lipid peroxidation within the body, indirectly reflecting cellular injury levels. CAT, meanwhile, mitigates oxidative damage by catalyzing the disproportionation of H_2_O_2_ [46], thereby decreasing hydroxyl radical content. SOD, GPX, CAT, and MDA constitute pivotal molecules in oxidative stress regulation [43]. Notably, icariin effectively enhances SOD, CAT, and GPX levels while suppressing MDA, achieved through modulation of the Keap1-Nrf2/HO-1 signaling axis. Consequently, icariin ameliorates oxidative stress imbalances in diabetes mellitus and confers renal protection [21]. (4) Modulating autophagy, a highly conserved catabolic mechanism that degrades superfluous or dysfunctional organelles and protein aggregates within lysosomes, represents a pivotal aspect in DN pathogenesis [47]. Prior investigations have highlighted impaired autophagy as a critical factor in DN development [48], suggesting that restoring autophagy activity may serve as a strategic therapeutic target for managing DN [22]. Broadly, LC3-II expression serves as a canonical marker for autophagosome formation in cellular and animal models [49]. Icariin promotes autophagy in renal tissues by upregulating LC3-II and downregulating p62 [22]. Furthermore, targeting the PI3K/Akt/mTOR signaling cascade, represents a viable strategy to restore autophagy and mitigate DN progression [50]. Icariin accomplishes this by attenuating the phosphorylation of PI3K, Akt, and mTOR, thereby fostering autophagy restoration [21].

### Limitations

(1) The limited number of included studies resulted in low statistical power; (2) High methodological heterogeneity, where variations in study design (e.g., dosing regimens, outcome measures) may lead to asymmetry; (3) The overall quality of the included randomized controlled trials was generally low, primarily due to inadequate reporting. Performance bias caused by a lack of blinding may overestimate treatment effects, while selection bias due to unclear randomization may compromise baseline comparability; (4) All included studies focused solely on non-toxicological investigations, omitting toxicity assessments; (5) The geographic concentration of the studies may limit generalizability to other populations or settings, necessitating multinational replication studies to validate these findings in the future; (6) Publication bias was present, as researchers may have preferentially published favorable results, leading to underrepresentation of negative studies; (7) No Kappa statistic was applied during data extraction to assess inter-rater reliability.

### Suggestions

(1) Enhance the comprehensiveness of research in this domain by augmenting safety and toxicity evaluations; (2) Comply with the reporting protocols stipulated by the Toxicology Society, accurately disclose whether the blind method is used; (3) Adopt forward-thinking registration practices for animal experiments(such as www.preclinicaltrials.eu or www.animalstudyregistry.org) to mitigate publication bias; (4) Truthfully report the negative results, so that the negative results can be accepted by more people; (5) Uphold high reporting standards in preclinical studies by adhering to frameworks such as ARRIVE guidelines [51] or HARRP [52] preclinical study reporting standards.

## Conclusion

In essence, our meta-analysis indicates that ICA exhibits potential in ameliorating DN through anti-inflammatory, antioxidant, and blood lipid-regulating mechanisms. Nevertheless, the methodological limitations and potential publication biases inherent in the included studies diminish the robustness of our conclusions. To validate and refine our findings, future endeavors should endeavor towards conducting large-scale, rigorously designed randomized controlled trials.

## Supplementary Information

Additional file 1.

Additional file 2.

## Data Availability

No datasets were generated or analysed during the current study.

## Abbreviations

ICA: Icariin
ARB: Angiotensin II receptor blockers
DM: Diabetes mellitus
DN: Diabetic nephropathy
ESRD: End-stage renal disease
SCR: Serum creatinine
BUN: Blood urea nitrogen
24 h UP: 24-Hour urinary protein excretion
24 h UV: 24-Hour urinary volume excretion
KI: Kidney index
MDA: Malondialdehyde
SOD: Superoxide dismutase
GPX: Glutathione peroxidase
ROS: Reactive oxygen species
IL-1β: Interleukin 1β
TG: Triglyceride
TC: Total cholesterol
