# Supplementary material for: Preclinical evidence of the effect of icariin on diabetic nephropathy: a systematic review and meta-analysis
Source: Diabetol Metab Syndr. 2025 Jun 18;17:222. doi: 10.1186/s13098-025-01760-2 (PMC12175312; doi:10.1186/s13098-025-01760-2)
Supplement: Supplementary file 1 — Additional file 1. [file 13098_2025_1760_MOESM1_ESM.docx]

EMBASE


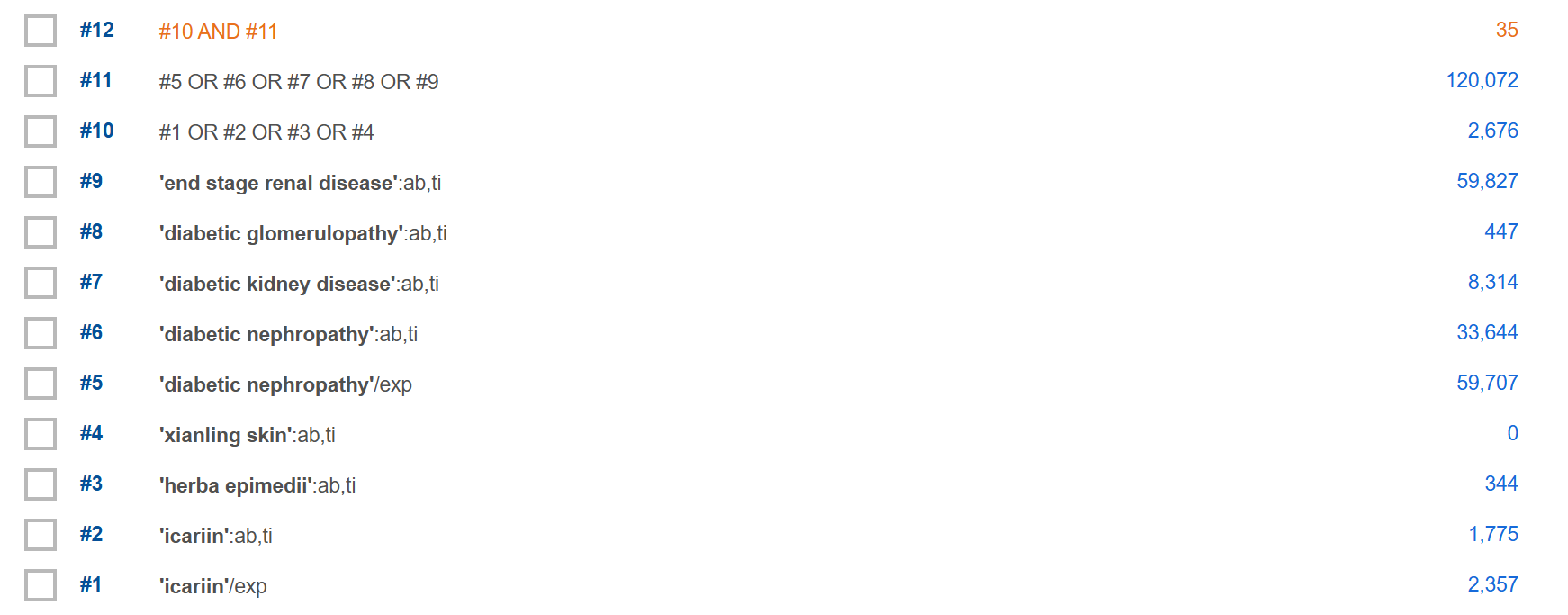


WOS

**
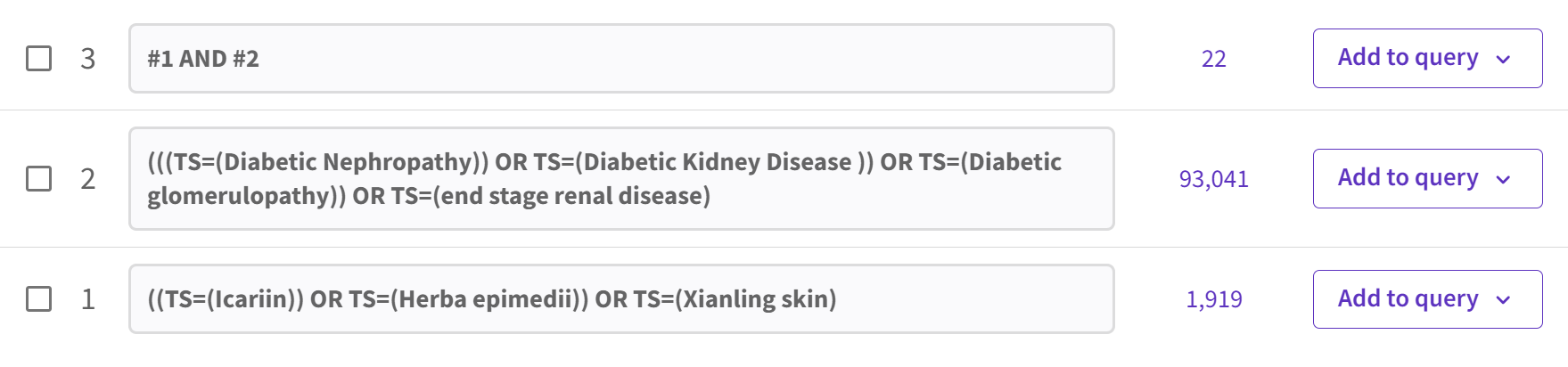
**

**PUBMED**


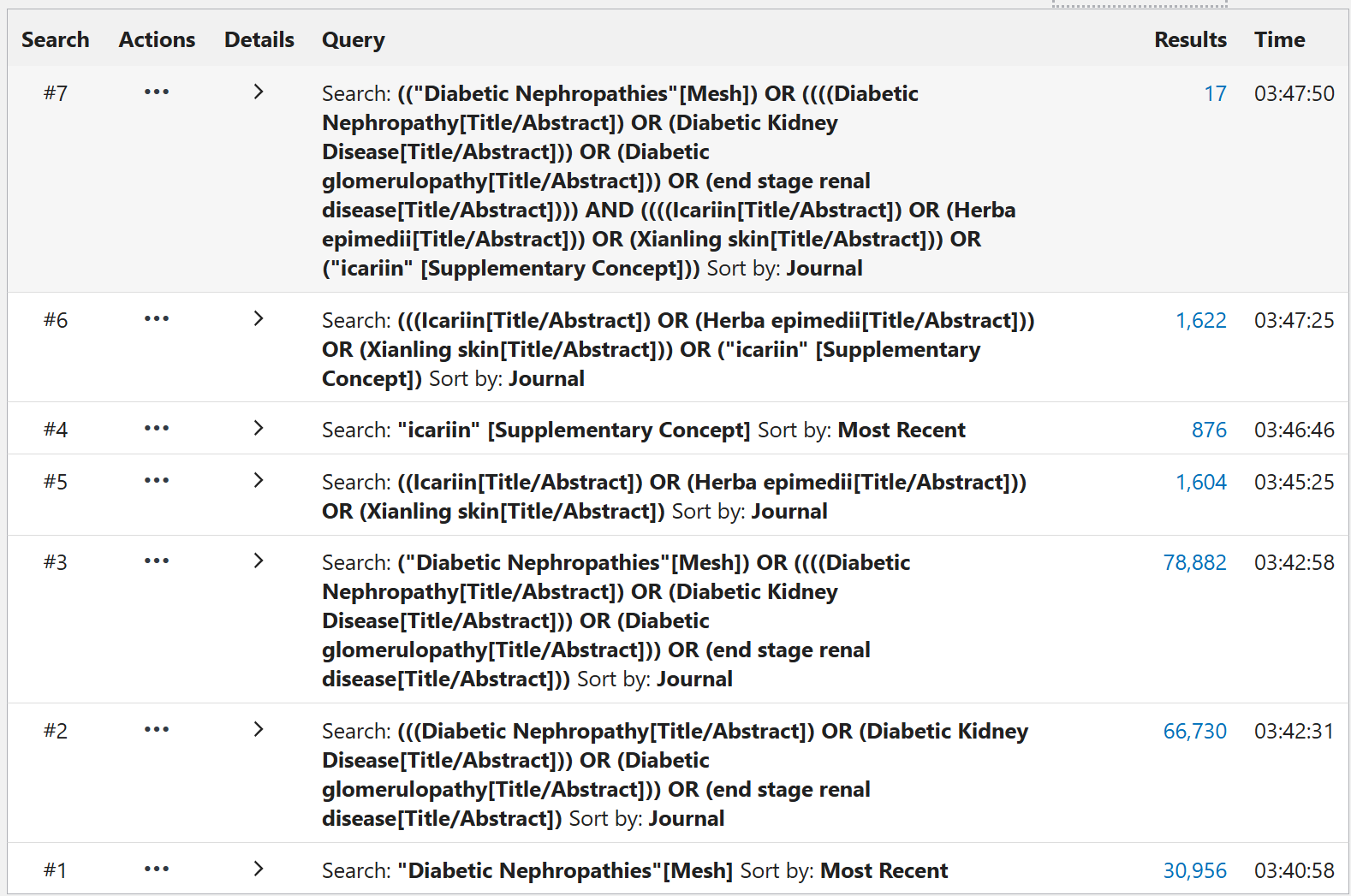


Cochrane


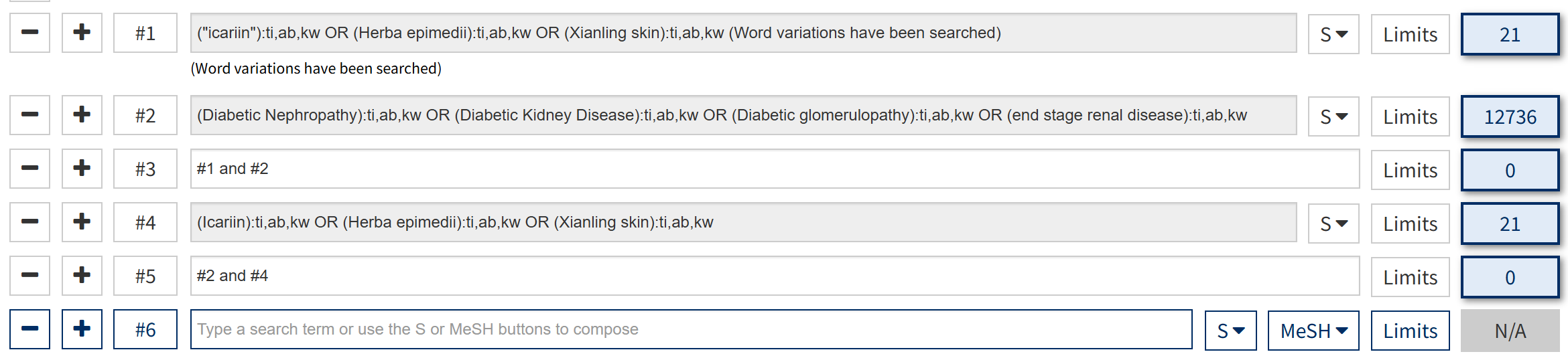


CBM


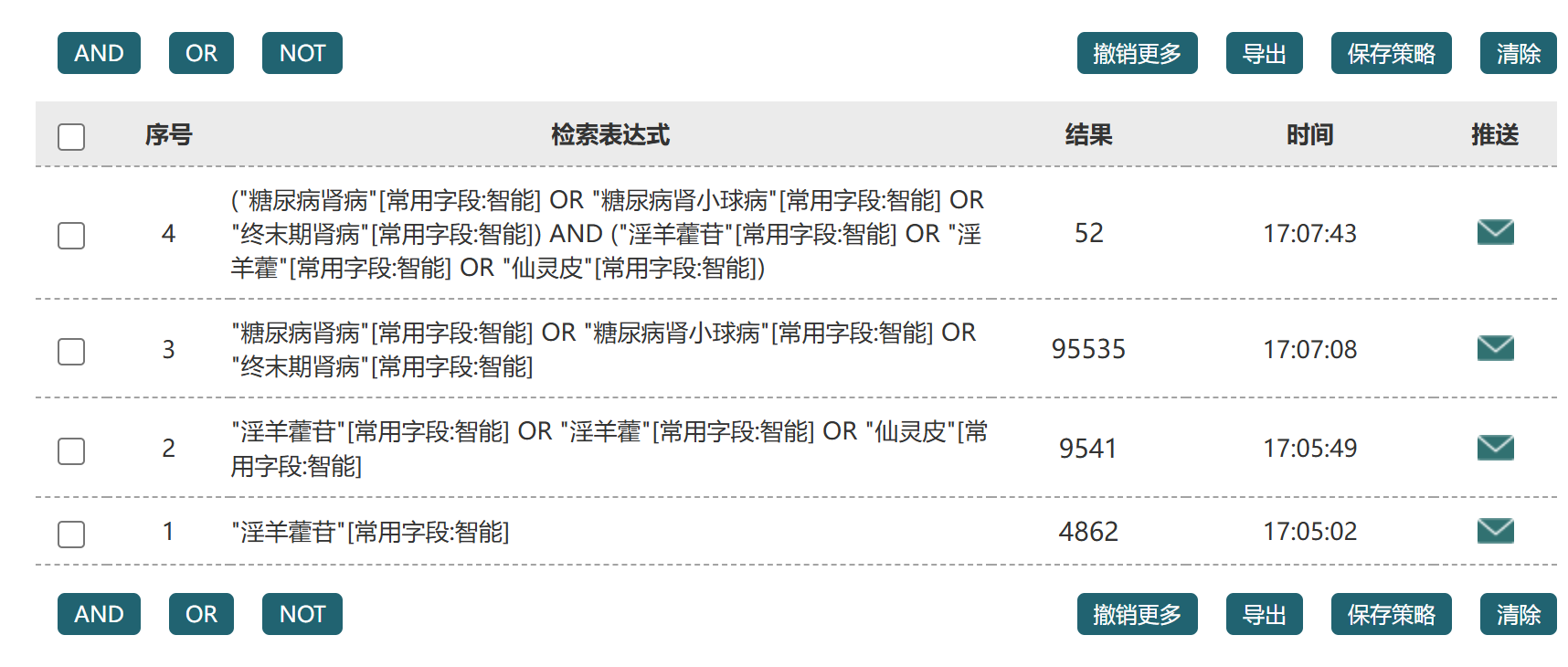


万方：

主题:(糖尿病肾病) and 主题:(淫羊藿苷)

知网：

（主题：糖尿病肾病）OR（主题：糖尿病肾小球病）OR（主题：终末期肾病）AND（主题：淫羊藿）OR（主题：淫羊藿苷）OR（主题：仙灵皮）
