# Supplementary material for: Preclinical evidence of the effect of icariin on diabetic nephropathy: a systematic review and meta-analysis
Source: Diabetol Metab Syndr. 2025 Jun 18;17:222. doi: 10.1186/s13098-025-01760-2 (PMC12175312; doi:10.1186/s13098-025-01760-2)
Supplement: Supplementary file 2 — Additional file 2. [file 13098_2025_1760_MOESM2_ESM.docx]

**Supplementary Material**

**Preclinical evidence of the effect of Icariin on diabetic nephropathy: A Systematic Review and Meta-Analysis**

**Diabetology & Metabolic Syndrome**

Xueli Man^1#^, Peiyao Ren^1#^, Juan Jin^1*^ and Qiang He^1*^

^1^ Department of Nephrology，the First Affiliated Hospital of Zhejiang Chinese Medical University (Zhejiang Provincial Hospital of Chinese Medicine)，Hangzhou, Zhejiang，310000, China

**Author’s contact information:**

^#^ for co-first author

Man Xueli: [Sherry_mang@163.com](mailto:Sherry_mang@163.com)

Ren Peiyao: [renpeiyao1997@163.com](mailto:renpeiyao1997@163.com)

^*^ for Corresponding Authors

Juan Jin, MD; Email: [lang_018@163.com](mailto:lang_018@163.com)

Qiang He, MD; Email: [gilsonmcff@gmail.com](mailto:gilsonmcff@gmail.com)

**Sensitivity analysis**

| Study | SMD (95% CI) | Weight% | P | I^2^ |
| --- | --- | --- | --- | --- |
| SCR | | | | |
| Original pooled | −2.18（−2.84，−1.53） | 100% | < 0.00001 | 78% |
| Excluding Chen 2012 | −2.28（−2.97，−1.58） | 6.2% | < 0.00001 | 79% |
| Excluding Cheng 2020 | −2.54（−3.29，−1.79） | 19.3% | < 0.00001 | 76% |
| Excluding Ding 2022 | −2.34（−3.11，−1.57） | 16.5% | < 0.00001 | 81% |
| Excluding Jia 2021 | −2.34（−3.08，−1.59） | 17.5% | < 0.00001 | 79% |
| Excluding Qi 2011 | −2.27（−2.97，−1.58） | 6.2% | < 0.00001 | 79% |
| Excluding Qi 2021 | −2.23（−2.92，−1.53） | 5.9% | < 0.00001 | 79% |
| Excluding Wang 2020 | −1.83（−2.46，−1.19） | 15.4% | < 0.00001 | 73% |
| Excluding Zang 2022 | −1.82（−2.42，−1.23） | 8% | < 0.00001 | 74% |
| Excluding Zhao 2020 | −2.08（−2.74，−1.43） | 5.2% | < 0.00001 | 77% |
| BUN | | | | |
| Original pooled | −2.45（−3.13，−1.78） | 100% | < 0.00001 | 77% |
| Excluding Chen 2012 | −2.55（−3.27，−1.83） | 6.3% | < 0.00001 | 78% |
| Excluding Cheng 2020 | −2.22（−2.84，−1.60） | 16% | < 0.00001 | 67% |
| Excluding Ding 2022 | −2.10（−2.72，−1.49） | 8.2% | < 0.00001 | 73% |
| Excluding Jia 2021 | −2.74（−3.53，−1.94） | 18.7% | < 0.00001 | 78% |
| Excluding Qi 2011 | −2.55（−3.27，−1.83） | 6.3% | < 0.00001 | 78% |
| Excluding Qi 2021 | −2.51（−3.23，−1.80） | 6.1% | < 0.00001 | 78% |
| Excluding Wang 2020 | −2.67（−3.48，−1.85） | 19% | < 0.00001 | 79% |
| Excluding Zang 2022 | −2.29（−3.01，−1.58） | 13.2% | < 0.00001 | 78% |
| Excluding Zhao 2020 | −2.51（−3.23，−1.79） | 6.3% | < 0.00001 | 78% |
| 24 UP | | | | |
| Original pooled | −2.20（−3.05，−1.36） | 100% | < 0.00001 | 72% |
| Excluding Ding 2022 | −1.57（−2.13，−1.02） | 14.7% | < 0.00001 | 40% |
| Excluding Jia 2021 | −2.98（−4.26，−1.70） | 37.1% | < 0.00001 | 77% |
| Excluding Qi 2021 | −2.38（−3.35，−1.41） | 12.3% | < 0.00001 | 75% |
| Excluding Wang 2020 | −2.39（−3.61，−1.17） | 35.9% | < 0.00001 | 77% |
| KI | | | | |
| Original pooled | −2.04（−2.80，−1.28） | 100% | < 0.00001 | 69% |
| Excluding Ding 2022 | −1.82（−2.70，−0.95） | 24.7% | < 0.00001 | 73% |
| Excluding Jia 2021 | −1.62（−2.38，−0.86） | 26.9% | < 0.00001 | 61% |
| Excluding Qi 2021 | −2.11（−2.97，−1.25） | 11.2% | < 0.00001 | 73% |
| Excluding Wang 2020 | −2.61（−3.49，−1.73） | 37.2% | < 0.00001 | 53% |

**Table 8.** Subgroup analysis for renal function indices according to animal model

| Subgroups/Outcomes | STZ | | |  | HFD+STZ | | |  | Subgroup  difference |
| --- | --- | --- | --- | --- | --- | --- | --- | --- | --- |
|  | SMD | P | I^2^ |  | SMD | P | I^2^ |  | P |
| SCR | -2.22 [-2.97, -1.47] | < 0.00001 | 78% |  | -2.10 [-3.66, -0.53] | 0.009 | 83% |  | < 0.00001 |
| BUN | -2.84 [-3.70, -1.97] | < 0.00001 | 80% |  | -1.53 [-2.35, -0.70] | 0.0003 | 52% |  | < 0.00001 |
| 24h UP | -2.98 [-4.26, -1.70] | < 0.00001 | 77% |  | -1.18 [-1.81, -0.55] | 0.0003 | 0% |  | < 0.00001 |
| KI | -1.62 [-2.38, -0.86] | < 0.0001 | 61% |  | -3.12 [-4.94, -1.29] | 0.0008 | 73% |  | < 0.00001 |

**Table 9.** Subgroup analysis for renal function indices according to animal gender

| Subgroups/Outcomes | Male | | |  | Female | | |  | Subgroup  difference |
| --- | --- | --- | --- | --- | --- | --- | --- | --- | --- |
|  | SMD | P | I^2^ |  | SMD | P | I^2^ |  | P |
| SCR | -2.20 [-2.97, -1.43] | < 0.00001 | 72% |  | -2.15 [-3.45, -0.86] | 0.001 | 86% |  | < 0.00001 |
| BUN | -2.41 [-3.18, -1.64] | < 0.00001 | 69% |  | -2.51 [-3.89, -1.13] | 0.0004 | 87% |  | < 0.00001 |
| 24h UP | -2.39 [-3.61, -1.17] | 0.0001 | 77% |  | -2.13 [-3.31, -0.96] | 0.0004 | 67% |  | < 0.00001 |
| KI | -2.61 [-3.49, -1.73] | < 0.00001 | 53% |  | -0.97 [-1.77, -0.18] | 0.02 | 52% |  | < 0.00001 |
